# Phytochemistry, pharmacological effects and mechanism of action of volatile oil from *Panax ginseng* C.A.Mey: a review

**DOI:** 10.3389/fphar.2024.1436624

**Published:** 2024-08-13

**Authors:** Yanan Xu, Shuai Bian, LiYing Shang, Xin Wang, Xueyuan Bai, Wei Zhang

**Affiliations:** ^1^ Northeast Asia Research Institute of Traditional Chinese Medicine, Changchun University of Chinese Medicine, Changchun, China; ^2^ Liangzhu Laboratory, Zhejiang University, Hangzhou, Zhejiang, China

**Keywords:** *P. ginseng* volatile oil, Phytochemistry, pharmacological effect, polyacetylenol, mechanism of action

## Abstract

*Panax ginseng* (*P. ginseng*), a traditional and highly valued botanical drug, has been used for thousands of years and is known around the world for its uses in food, medicine, and healthcare. The comprehensive study of *P. ginseng* is crucial for the quality assurance of medicinal materials and optimal resource utilization. Despite being present in trace amounts, *P. ginseng* volatile oil has a wide range of chemical metabolites with important medicinal potential. The volatile oil has shown promise in defending the cardiovascular system, as well as in terms of its ability of antibacterial, anti-aging, anti-platelet coagulation, anti-inflammatory, support the nervous system nutritionally, and shield it from harm. Due to its low composition and lack of thorough investigation, *P. ginseng* volatile oil’s therapeutic applicability is still restricted although it exhibited many benefits. This review aims to provide insights into the chemical composition, extraction processes, pharmacological effects, and mechanisms of action of *P. ginseng* volatile oil, and to provide theoretical support and guidelines for future research and clinical application.

## 1 Introduction

*Panax ginseng* C.A.Mey. [Araliaceae, Ginseng radix et rhizoma], as a traditional botanical drug with a long history, has occupied a pivotal position in Chinese traditional medicine since ancient times. *P. ginseng* has a slightly bitter and warm taste, serving as a great tonic that can boost energy, restore the pulse, nourish the spleen and lungs, replenish blood, calm nerves, and improve intelligence (National Pharmacopoeia Commission, 2020). With the development of modern science and technology, more medicinal values of *P. ginseng* have been gradually explored by researchers around the world, and its application fields have been expanded from food and health products to the medical field (Mancuso and Santangelo, 2017). More than 300 active metabolites, including polysaccharides, ginseng peptides, ginsenosides, flavonoids (Liu et al., 2020), volatile oils, organic acids, alkaloids, trace elements, and vitamins, have been identified (Su et al., 2023). The medicinal value of *P. ginseng* is not only reflected in its polysaccharides, ginsenosides and other metabolites, volatile oil as one of the metabolites. Although it only accounts for about 0.02%–2.5% (Peng et al., 2017; Sun et al., 1993), its biological activity cannot be ignored. *P. ginseng* volatile oil (GVO) has shown significant efficacy in cardiovascular protection, antimicrobial, anti-aging, anti-platelet aggregation, anti-inflammatory, nutritional support, and neurocellular protection. The diversity of its chemical metabolites and the potential of its pharmacological activities provide a wide scope for future developmental studies.

**FIGURE 1:**
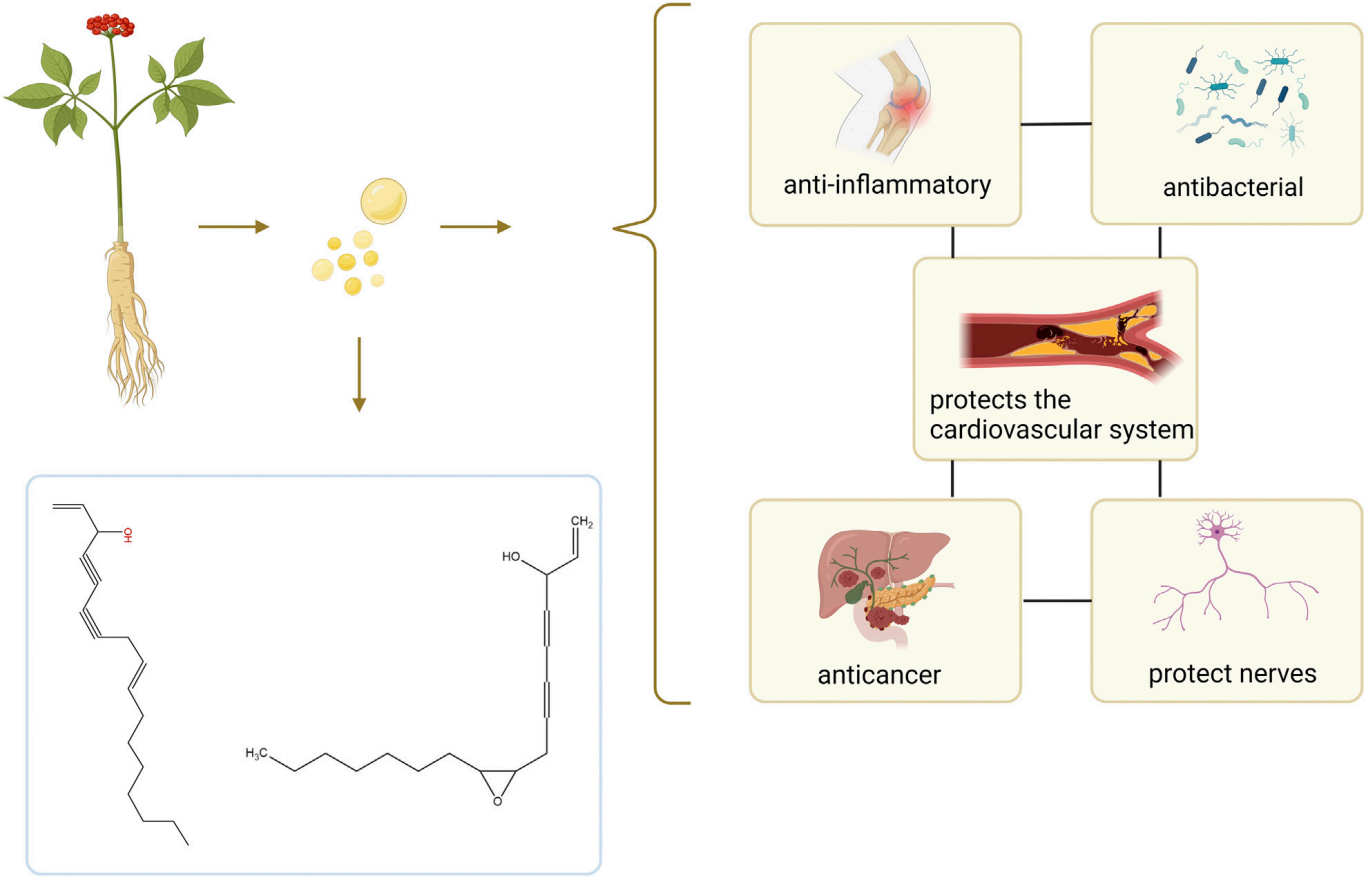
Summary of the composition and effect of GVO. GVO has a special aroma, and its main metabolites are Linoleic acid, panaxynol, panaxydol and so on. It has anti-inflammatory, antioxidant, anti-aging, anti-cancer, neuroprotective, and fatigue-relieving properties.

In recent years, relatively little research has been conducted on the volatile oil of ginseng, and limited data are available for reference. This has prompted us to conduct a more comprehensive and in-depth exploration of it. In this paper, we will analyze the chemical composition of GVO, discuss the volatile oil metabolites extracted from different parts of *P. ginseng* and their pharmacological effects, and also investigate the effects of different regions and years of growth on the volatile oil content of *P. ginseng*. The summary was further refined with the help of the newidea.ai (https://www.newidea.ai/home). In terms of the extraction process, this paper will compare the advantages and disadvantages of traditional and modern methods and explore their effects on the composition and medicinal effects of volatile oils. In terms of pharmacological effects and mechanisms of action, this paper will detail the effects of GVO on the cardiovascular system, anti-inflammatory, antibacterial, anticancer, etc., and explore its potential molecular targets and mechanisms of action (Figure 1). In summary, this paper will provide a comprehensive theoretical basis for the research and application of GVO and point out the direction for future development and utilization. Through in-depth exploration of the chemical composition, pharmacological activity and mechanism of action of GVO, we expect to be able to provide scientific support for the in-depth development and clinical application of *P. ginseng* resources.

## 2 The phytochemical composition of GVO

**FIGURE 2:**
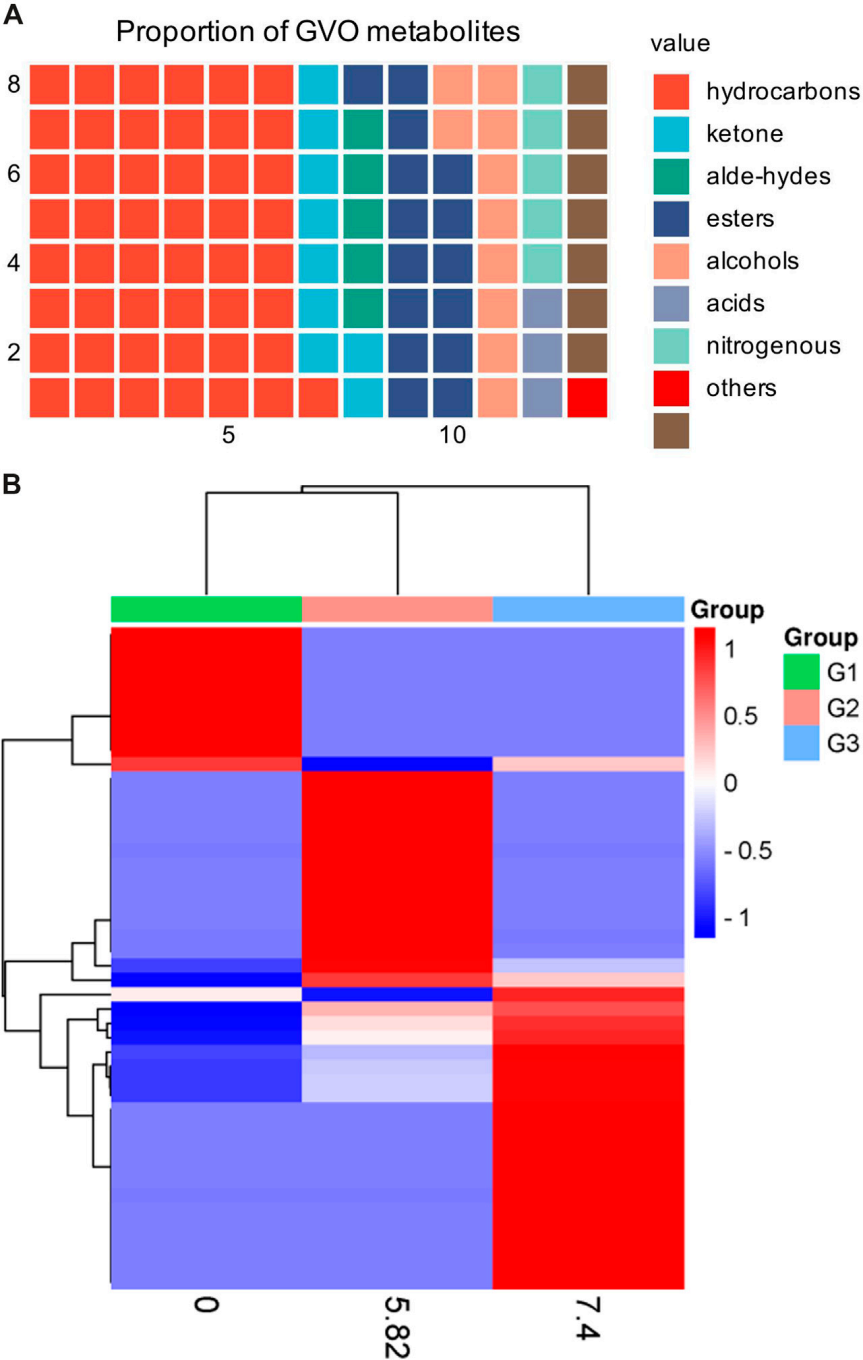
The main metabolites of GVO and its differences under diverse processing conditions. **(A)** Proportion of different types of metabolites of GVO. **(B)** Heat map of chemical composition differences among fresh ginseng G1, white ginseng G2, and red ginseng G3.

GVO possesses a distinct aroma. The *P. ginseng* aroma developed through organic culture method (OCM) and GAP method exhibited the highest levels of beet saponin and aromatic alkene, which are recognized as key metabolites of the *P. ginseng* aroma (Lee et al., 2012). In research, GC-MS is commonly utilized to assess the composition and quality of volatile oils (Daferera et al., 2000). Through this method, modern scholars have identified 369 metabolites in GVO, comprising 154 hydrocarbons, 35 ketones, two aldehydes, 55 esters, 37 alcohols, 12 acids, 22 nitrogen-containing metabolites, and 52 other metabolites (Qiu et al., 2008), as shown in Figure 2; Table 1. Among the volatile metabolites of *P. ginseng*, closely related to the aroma properties of the plant are sesquiterpenoids, accounting for about 40% of the volatile oil, followed by panaxynol and panaxydol (Cho et al., 2012). Currently, over 20 types of polyacetylene derivatives have been isolated from *P. ginseng* (Yeo et al., 2017), including panaxynol, panaxydol, panaxydiol, panaxytriol, panaxacol, panaxyne epoxide, ginsenoyne A− K. Recently, new polyacetylenes metabolites with carbonyl group replacing the hydroxyl group had been isolated such as 9,10-epoxyheptadecan-4,6-diyn-3-one, one-ethoxy-9,10-epoxyheptadecan-4,6-diyn-3-one and 9,10-epoxy-16-heptadecan-4,6-diyn-3-one.

**TABLE 1:** Partial metabolites of GVO.

Categorization	Metabolite name	Molecular formula	CAS
Terpenoid	β-Guaiene	C_15_H_24_	88-84-6
	β-patchouline	C_15_H_24_	514-51-2
	β-farnesene	C_15_H_24_	18794-84-8
	NNNb-elemene	C_15_H_24_	251-713-0
	α-cubebene	C_15_H_24_	17699-14-8
	zingiberene	C_15_H_24_	495-60-3
	A-Neoclovene	C_15_H_24_	4545-68-0
	Humulene	C_15_H_24_	6573-98-6
	Sqalene	C_30_H_50_	7683-64-9
	caryophyllene	C_15_H_24_	13877-93-5
	β-caryophllene	C_15_H_24_	87-44-5
Alcohols	sulfole160	C_16_H_34_S	25360-09-2
	ergostenol	C_28_H_48_O	632-32-6
	Chondrillast-7-enol	C_29_H_50_O	18525-35-4
	hydroxy steroids	C_29_H_48_O	68555-08-8
	β-sitosterol	C_29_H_50_O	5779-62-4
	panaxynol	C_17_H_24_O	81203-57-8
	panaxydol	C_17_H_24_O_2_	72800-72-7
	panaxydiol	C_17_H_24_O_2_	63910-76-9
	(-)-panaxytriol	C_17_H_24_O_3_	87005-03-6
Ketones, aldehydes	β-saccharostenone	C_29_H_46_O	2034-72-2
	β-sitostenone	C_29_H_48_O	1058-61-3
	pacoch3	C_17_H_34_O	2922-51-2
	octylaldehydes	C_8_H_16_O	124-13-0
Phenols, heterocyclics	4-ethenyl-2-methoxyphenol	C_9_H_10_O_2_	7786-61-0
	vita plus E	C_29_H_50_O_2_	59-02-9
	n-hexatricontane	C_36_H_74_	630-06-8
	octatriacontane	C_38_H_78_	7194-85-6
	n-henicosane	C_21_H_44_	629-94-7
	alkane c15	C_15_H_32_	629-62-9
	1, 2-hexadecene epoxide	C_16_H_32_O	7320-37-8

### 2.1 A chemical analysis of the volatile oils extracted from different parts of the *P. ginseng* plant

#### 2.1.1 Volatile oil metabolites in flower buds

**FIGURE 3:**
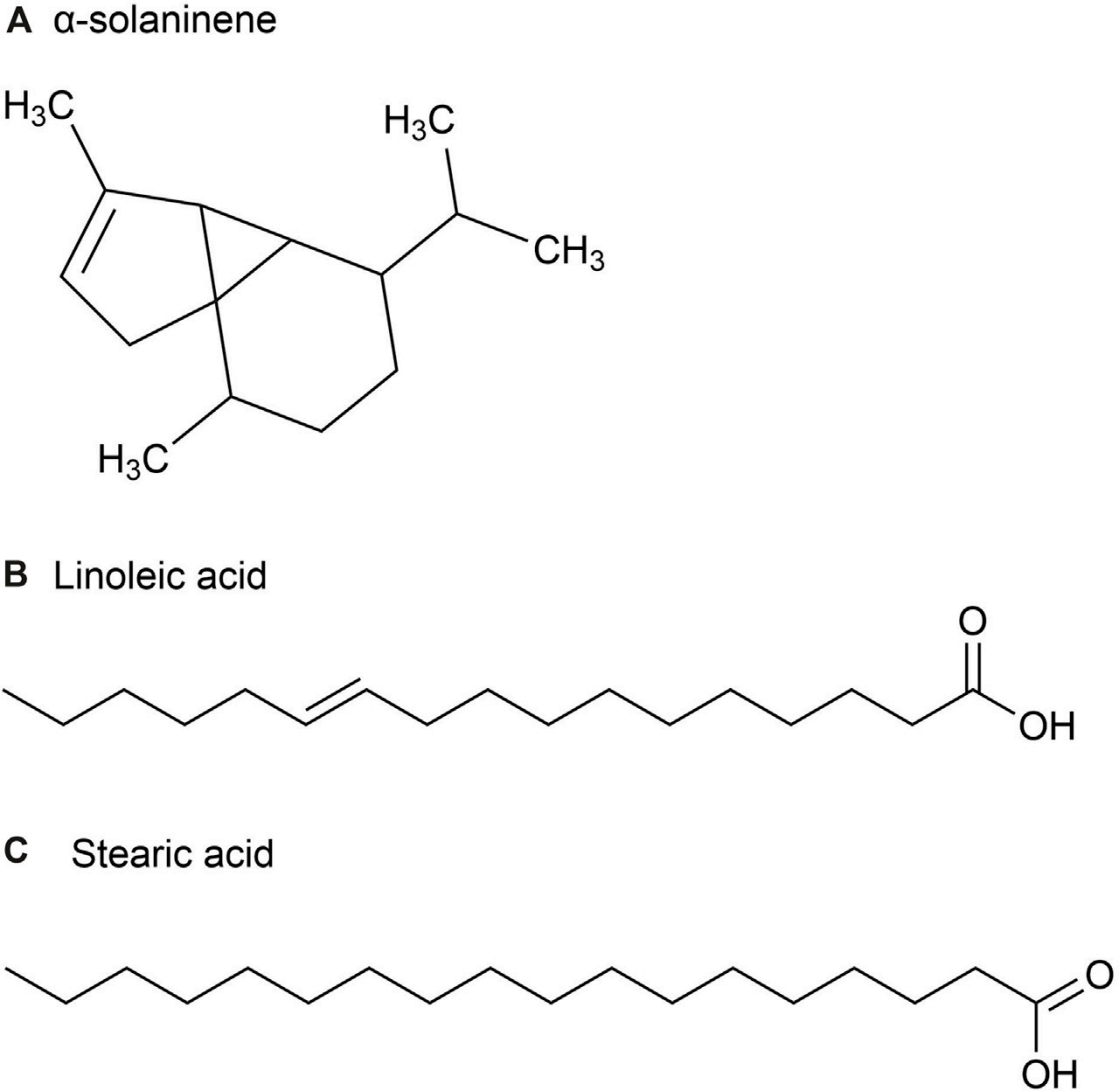
The main metabolites of GVO in flower buds. **(A)** α-solaninene. **(B)** Linoleic acid. **(C)** Stearic acid.

The volatile oil content of *P. ginseng* varies across different parts of the plant. Although *P. ginseng* flowers (GFs) buds are not recorded in the Chinese Pharmacopoeia (2020 edition), GFs are also non-traditional medicinal parts with anti-fatigue and immune-enhancing properties. Studies have shown that the composition of volatile oils in *P. ginseng* flowers generally remains consistent over time (Mao et al., 1989). The volatile oil extracted from it is a light yellow transparent oily liquid with a yield of about 0.2%. After identification, 23 chemical metabolites were identified from 51 chromatographic peaks, including 10 sesquiterpenes, eight alkanes, 2 esters, and one ketone, accounting for 43.5%, 34.8%, 8.7%, 8.796, and 4.3% of the total volatile oils, respectively (Figure 3). Among the sesquiterpenes, α-solaninene (Figure 3A), α-sandalpinene, β-sandalene and (3*Z,*6*E*)-α-farnesene were discovered for the first time.

Furthermore, some scholars have used GC-MS analysis to identify 45 chemical metabolites in the volatile oil of *P. ginseng* flowers (Ma et al., 1992). Among them, the highest content is Linoleic acid (37.06%) (Figure 3B), followed by Stearic acid (17.77%) (Figure 3C). Other main metabolites include aldehydes, enals, unsaturated alcohols, higher alkanes, alkynes, etc. Distribution density and accumulation of oil cells affect volatile oil content. Therefore, *P. ginseng* flowers may contain volatile oils that are closely related to oil cell growth and development (McAdam et al., 2020). As *P. ginseng* blooms after 3 years, its early accumulation of substances is deeper, providing more energy and nutrients to oil cells. Consequently, the volatile oil content is the highest in three-year-old *P. ginseng* flowers (Du et al., 2023).

#### 2.1.2 Volatile oil metabolites in stems and leaves

Traditionally, *P. ginseng* leaves have been utilized in China as a medicine for treating diseases. In comparison to *P. ginseng* roots, its leaves have a shorter growth period and lower cost, making them both economically and medicinally valuable. However, little attention has been given to the chemical composition of the volatile oil in *P. ginseng* leaves. In a study, *P. ginseng* leaves and stems were extracted, yielding a black-green crystalline volatile oil of 0.14% (Liu et al., 2002).

**FIGURE 4:**
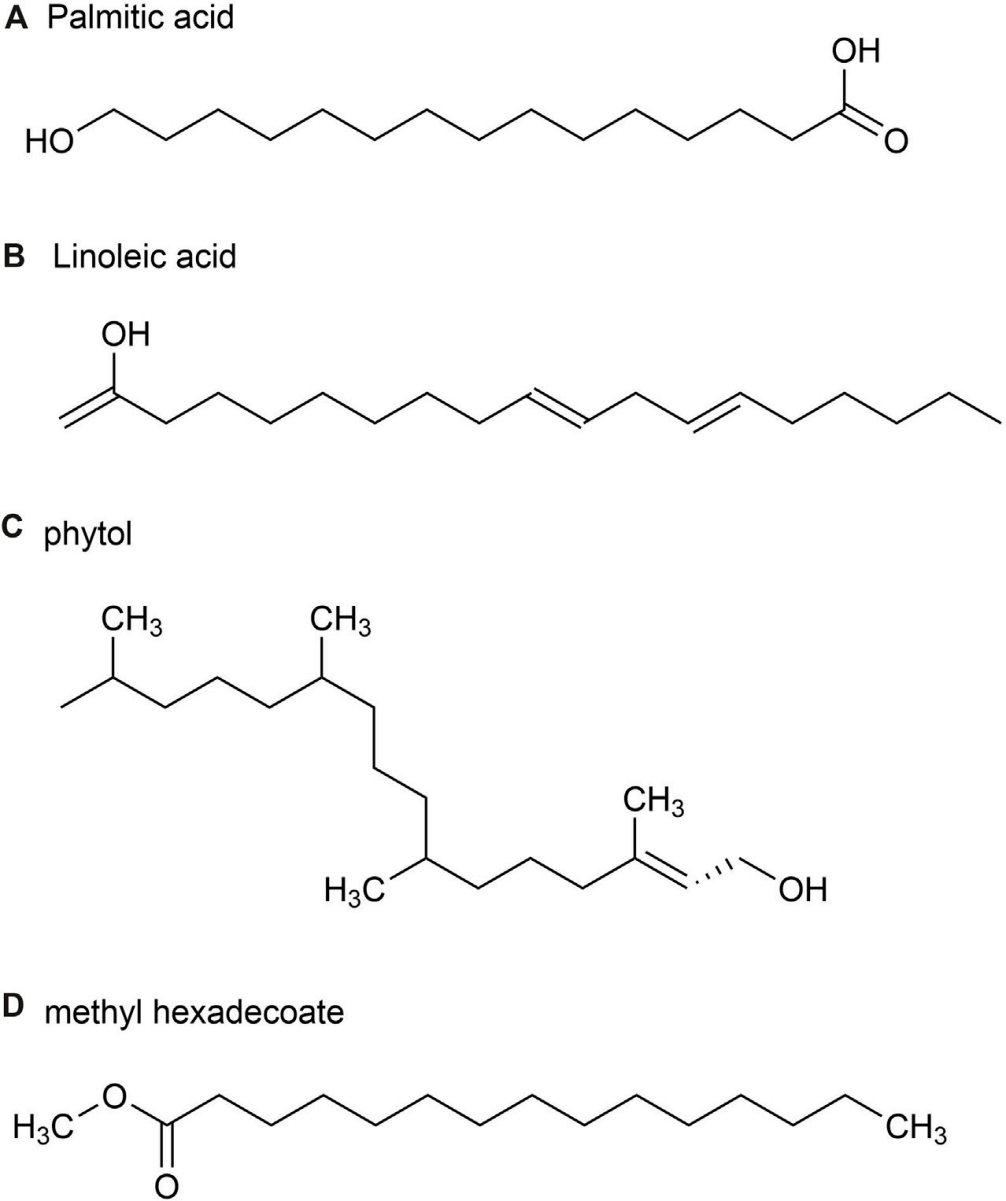
The main metabolites of GVO in stems and leaves. **(A)** palmitic acid. **(B)** Linoleic acid. **(C)** phytol. **(D)** methyl hexadecoate.

A total of 54 metabolites were identified, predominantly consisting of aliphatic (69.0%), terpenoids (21.5%) and aromatic (2.4%). The major metabolites identified in these parts include Palmitic acid (36.1%) (Figure 4A), followed by (*E*)-β-farnesene (15.4%), Linoleic acid (9.8%) (Figure 4B), phytol (5.6%) (Figure 4C) and methyl hexadecoate (2.9%) (Figure 4D). Sesquiterpene hydrocarbons accounted for 20.3%, while oxidized sesquiterpene hydrocarbons accounted for 0.6%, and monoterpene hydrocarbons accounted for 0.6% of terpenoids (Jiang et al., 2014).

#### 2.1.3 Volatile oil metabolites in fruits

**FIGURE 5:**
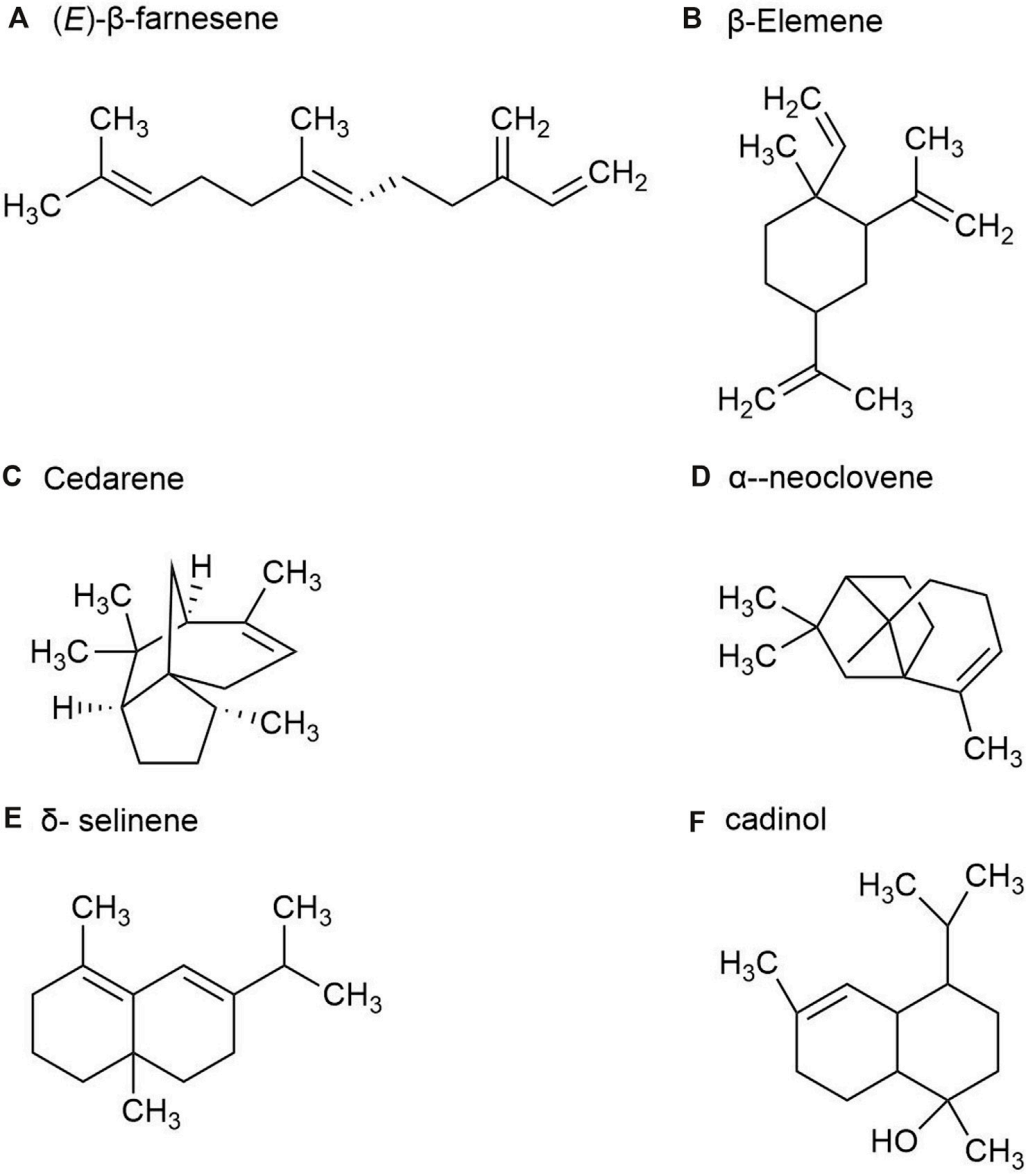
The main metabolites of GVO in fruits. **(A)** (E)-β-farnesene. **(B)** β-Elemene. **(C)** Cedarene. **(D)** α-neoclovene. **(E)** δ-selinene. **(F)** cadinol.

*P. ginseng* fruit is the dried ripe fruit of *P*. *ginseng*. Its chemical metabolites include ginsenoside, volatile oil, carbohydrate and sugar, amino acid and allaloids, vitamins and minerals. A total of 23 volatile metabolites, mainly composed of sesquiterpenes, have been identified from *P. ginseng* fruits of three different colors, red fruit, yellow fruit, and orange fruit, such as (*E*)-β-farnesene (Figure 5A), β*-*Elemene (Figure 5B), Santene, Cedarene (Figure 5C), and α-neoclovene (Figure 5D). The total sesquiterpene content of red fruits is the highest, followed by orange and yellow fruits, with significant differences between samples. Yellow fruits contain a significant amount of δ-selinene (Figure 5E), β-caryophyllene, *α*-farnesene ginsenosol and cadinol (Figure 5F). As a consequence, *P. ginseng* fruit has different volatile (Cui et al., 2020).

#### 2.1.4 Volatile oil metabolites in rhizomes

**FIGURE 6:**
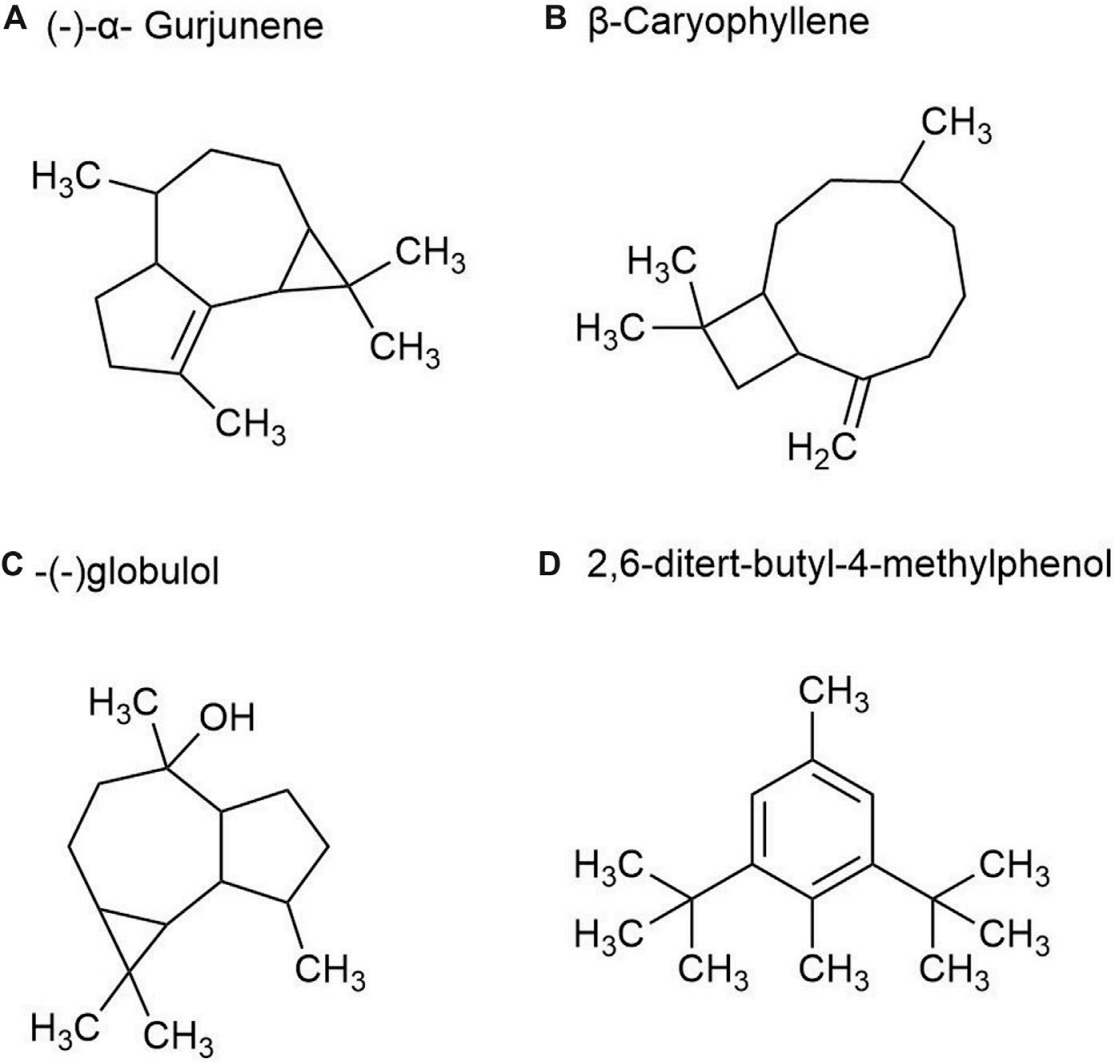
The main metabolites of GVO in rhizomes. **(A)** (-)-α- Gurjunene. **(B)** β-Caryophyllene. **(C)** -(-)globulol. **(D)** 2,6-ditert-butyl-4-methylphenol.

There are more than 40 kinds of chemical metabolites in the volatile oil of *P. ginseng* root, mainly including esters, monoterpenes, alkanes, and sesquiterpenes. As part of the sesquiterpene family, sesquiterpenoids are characteristic metabolites of GVO. Such as β*-*Ginsenene*,* (−)-α*-* Gurjunene (Figure 6A), β-Elemene*,* β*-*Caryophyllene (Figure 6B), β-New clove tricycline (molecular formula C_15_H_24_) and sesquiterpene oxygen-containing metabolites (mainly referring to alcohols such as spartanol, ginsenosol, and -(−)globulol (Figure 6C)) *α-*Juniperol, etc., with a molecular formula of C_15_H_24_O) (Richter et al., 2005; Ding, 2008). It was found that the content of total volatile oil in roots increased with the growth age of *P. ginseng*. A study was conducted by steam distillation to extract the volatile oil content in *P. ginseng* reeds, and the yield was 0.35%. The main differences with *P. ginseng* root were palmitic acid, 2,6-ditert-butyl-4-methylphenol (Figure 6D) and methyl octadenoate, with the contents of 2.08%, 1.80% and 1.44%, respectively (Zheng et al., 1989).

**FIGURE 7:**
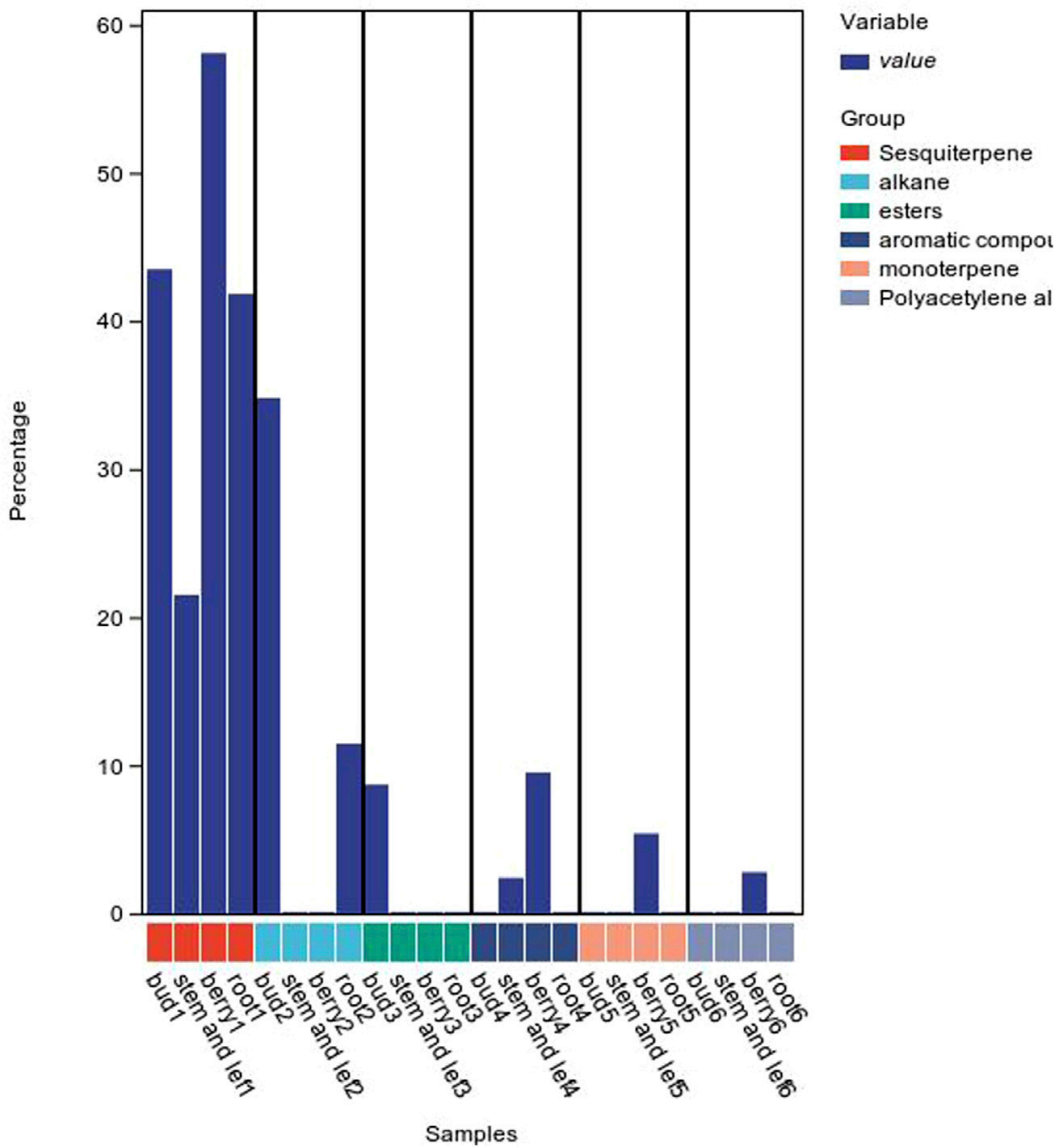
Volatile oil content of *P. ginseng* in different parts.

The composition of volatile oils from different parts of *P. ginseng* varies, as shown in Figure 7. Sesquiterpenes were the most abundant metabolites in flowers, followed by alkanes and esters. The stems and leaves contain sesquiterpenes, aromatic metabolites. The fruit of *P. ginseng* has the highest percentage of sesquiterpene content compared to other parts of the plant. In *P. ginseng* root, the main metabolites are sesquiterpenes and alkanes. In addition to this, there is also oil in *P. ginseng* seeds. *P. ginseng* seed oil is mainly composed of non-volatile fatty acids, followed by phenolic compounds (Zhu et al., 2010; Lee et al., 2013)

### 2.2 Effects of different regions on the volatile oil content of *P. ginseng*

**FIGURE 8:**
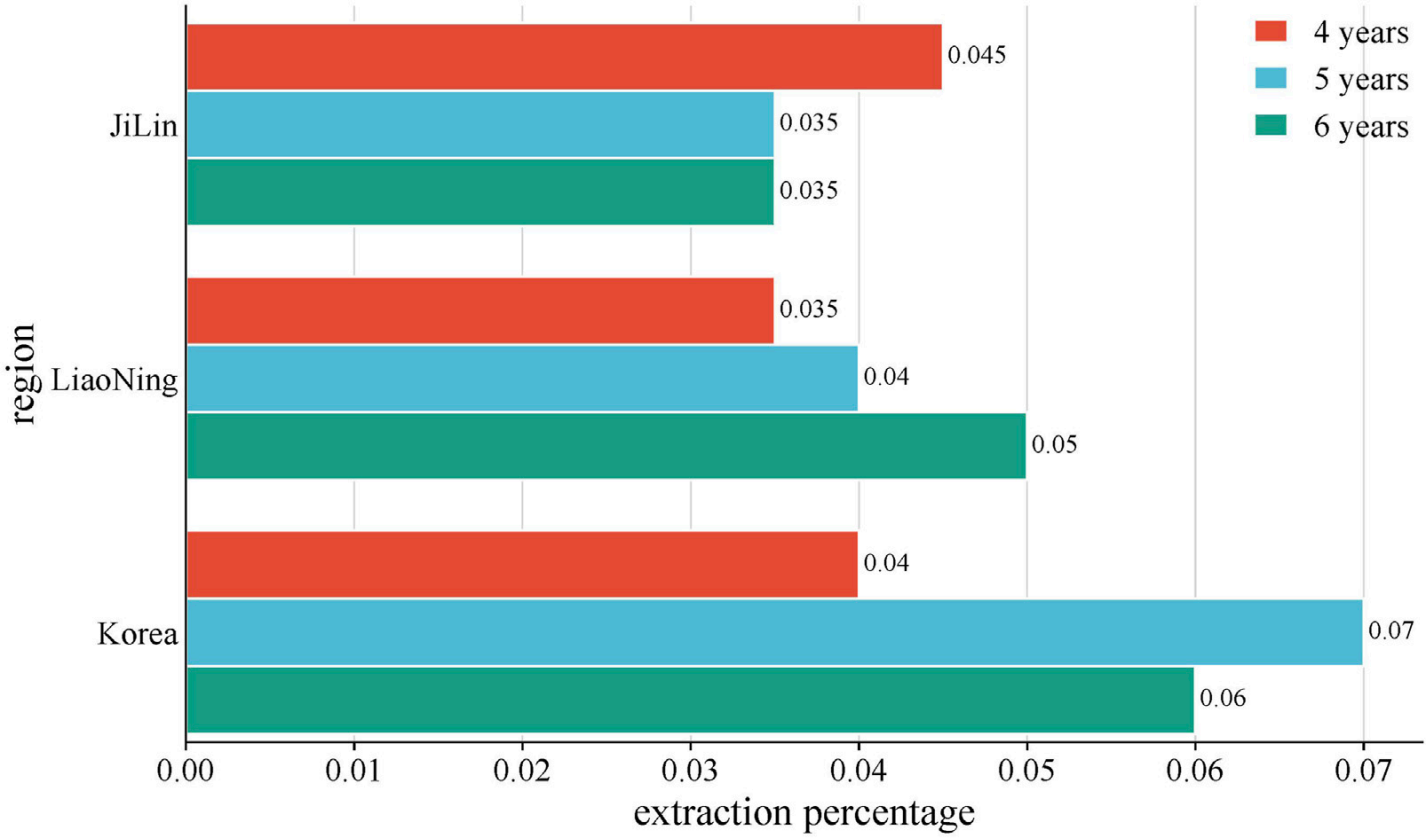
Average yield of GVO by region. The average volatile oil yield of *P. ginseng* from Jilin, Liaoning, and South Korea in China was compared with. It was found that the volatile oil content of four-year-old *P. ginseng*: Jilin Province > Liaoning Province > Korea. Five-year-old *P. ginseng*: Korea > Liaoning Province > Jilin Province. Six-year-old *P. ginseng*: Liaoning Province > Korea > Jilin Province.

The volatile oil composition and content of *P. ginseng* varied in different regions and years of growth, as shown in Figure 8. A study was conducted to compare and summarize the quality and yield of GVO from several counties under the provinces of Jilin, Liaoning, and Heilongjiang with those of Korean ginseng. Among the *P. ginseng*s of different ages and origins, the one with the highest volatile oil yield was the four-year-old Antu *P. ginseng*, and the one with the lowest yield was the six-year-old Jian *P. ginseng*. The mean value of the volatile oil yield of *P. ginseng* roots from all origins was 0.056%, with an RSD of 26%, indicating that the volatile oil content of *P. ginseng* differed significantly among different origins and ages (Wang, 2016).

Different growth forms of *P. ginseng* have varying levels of volatile oil content. The volatile oils of cultivated *P. ginseng* (CG), transplanted *P. ginseng* (TG) and mountain cultivated *P. ginseng* (MCG) were extracted by headspace solid-phase microextraction-gas chromatography-mass spectrometry, followed by chromatographic identification using n-alkane standard (C7-C30). Calculating and comparing the aldehydes, terpenes, alcohols, alkynes, esters, and other metabolites of three types of *P. ginseng*, it was found that the content of terpenoids was the highest, with CG (85.91%), MCG (90.27%), and TG (76.89%), respectively. However, a difference in alkyl alcohol content between *P. ginseng* samples of different origins was not statistically significant (Gu et al., 2022).

### 2.3 Effect of concoction on the volatile oil composition of *P. ginseng*

When *P. ginseng* is steamed and dried, it is produced as red ginseng. Research has indicated that the conversion of *P. ginseng* into red ginseng leads to a loss in total volatile oil content ranging from 63.89% to 74.54%, averaging 69.50% (Wu et al., 1992). In addition, the composition of volatile oil is altered during this process, as depicted in Figure 2B. The transformation of *P. ginseng* into red ginseng results in a change in the composition of GVO. The main metabolites of red ginseng oil (RGO) include linoleic acid, palmitic acid, β-sitosterol, γ-sitosterol, and stigmasterol, which are also present in GVO. The relative content of C_4_-C_6_ metabolites in red ginseng and fresh ginseng differs significantly, with fresh ginseng containing 1.04% of C_4_-C_6_ metabolites compared to red ginseng. Fresh ginseng contains three C_10_ monoterpenes, while red ginseng contains only one. The content of soy sterols and β-sitosterol also differed in red ginseng and *P. ginseng*. Notably, the content of stigmasterol in five-year-old and six-year-old red ginseng was reported to be 23.84 mg/g and 27.46 mg/g, respectively. The content of beta-sitosterol in five and six-year-old *P. ginseng* was 72.58 mg/g and 82.14 mg/g (Lee et al., 2018) respectively. In addition, a study comparing the volatile characteristics of fresh, white and red ginseng, found that fresh *P. ginseng* had a stronger odor than red ginseng (Abd El-Aty et al., 2008). The main functional groups identified in white and red ginseng were alcohols, ketones, esters, and phenols, with acids being found only in fresh *P. ginseng*. Therefore, it can be hypothesized that during the processing of fresh ginseng, many volatile metabolites may disappear or increase (Cho, 2015).

For different concoctions, the content of stigmasterol (metabolite 1) and beta-sitosterol (metabolite 2) in *P. ginseng* varied greatly from year to year. Of the three concocted forms, the total metabolite content of red ginseng was least affected by year. White *P. ginseng* showed the greatest variation in content and had the highest levels of metabolites in the six-year-old. So when it comes to experiments related to P. ginseng phytosterols, researchers need to choose according to their own experimental requirements (Lee et al., 2018).

Although there are differences in the composition of volatile oils derived from red ginseng and *P. ginseng*, their pharmacological effects, mechanism of action, targets and pathways are comparable. RGO has been found to possess antitumor activity (Lee et al., 2010). It inhibits tumor transformation and blocks the activation of NF-kB, AP-1, and MAPK, as well as the expression of COX-2 (Truong et al., 2018). This anticancer pathway is similar to that of GVO. β-sitosterol and linoleic acid (Yasuda et al., 2009) present in RGO have been identified as effective substances with anti-tumor and neuroprotective properties (Lee et al., 2017). β-Sitosterol promotes cell cycle arrest and apoptosis in breast cancer cells (Vundru et al., 2013), prostate cancer cells (von Holtz et al., 1998), and inhibits proliferation of human gastric adenocarcinoma cells and xenograft tumors. Furthermore, RGO also has anti-inflammatory effects (Bak et al., 2012a), which can significantly reduce the serum levels of NO, IL-6 and TNF-a in mice, as well as the expression of colon inflammation markers iNOS, COX-2, IL-6, IL-1β and TNF-α (Truong et al., 2019). Similarly, GVO can also reduce the aforementioned inflammatory factors in the serum to achieve anti-inflammatory effects.

Red ginseng, exhibits higher antioxidant activity due to the increase in phenolic metabolites induced by steam during the preparation process (Kang et al., 2006). RGO can effectively inhibit DPPH and ABTS free radicals. It may also significantly reduce the levels of liver enzymes (ALT and AST) in the serum of mice, increase the levels of antioxidant enzymes (SOD and CAT), reduce the content of DNA oxidation products (8-OHdG) (Ullah et al., 2021), and protect the liver from oxidative stress. Moreover, red ginseng oil also directly scavenges ROS (Meerson et al., 1982), inhibits lipid peroxidation, and protects cells from oxidative damage by inhibiting the MAPK signaling pathway to induce the expression of cellular antioxidant enzyme activity (Abe and Berk, 1998). In addition, RGO also has antibacterial effects (Reyes et al., 2017) and has the ability to control acne. It promotes anti-melanin production (Saba et al., 2020), hair growth, and protects the skin from UVC radiation (Truong et al., 2021). GVO is similar to red GVO chemical composition. However, during the processing of red ginseng, some metabolites are lost while new substances are produced. Both types of volatile oil exhibit similar pharmacological effects, but further comparative studies are necessary to determine which one yields superior results.

### 2.4 Effects of different growth years on the volatile oil content of *P. ginseng*

The volatile oil content of *P. ginseng* varies with the plant’s age, generally showing an increasing trend as the ages. The older the *P. ginseng*, the better its quality, mainly due to the accumulation of active metabolites with age. Research has found that the relative abundance of a-cadinol, a-bisabolol, thujob-sene, and n-hexadecanoic acids in volatile oils increases most significantly. By comparing the relative amounts of these metabolites, the quality of GVO can be evaluated (Qiu et al., 2008).

Principal metabolite analysis (PCA) was performed on the volatile oil of *P. ginseng* during the third, fifth and eighth year growth periods, and it was found that there were significant differences in the volatile oil of different years. In particular, the samples of groups 7, 8 and 9 had obvious dispersion compared with other groups, which proved that there was a significant difference in the composition of eight-year-old *P. ginseng* compared to samples of other ages. The spots on samples 1, 2, and three are located in smaller areas, indicating that the chemical composition differences of the samples over the past 3 years are relatively small.

Samples 1, 2, and three all have spots located in smaller areas, indicating that there is very little difference in chemical composition between the samples over the course of the past 3 years.

## 3 Extraction process of GVO

The volatile oil of *P. ginseng* is composed of various metabolites with low content, solubility, and boiling points, as well as highly unstable properties. Therefore, the efficiency and rationality of the extraction method are crucial. Volatile oil extraction methods can be classified into traditional and innovative methods. Traditional methods include steam distillation, impregnation, infiltration, and reflux extraction. With technological advancements, new methods such as ultrasonic extraction, microwave extraction, semi-biomimetic extraction, and solid phase microextraction have been developed (Table 2). Among the available techniques, supercritical fluid extraction technology offers a higher extraction rate and less pollution, although it is not suitable for large-scale production. The composition of volatile oils from traditional Chinese medicine can vary based on the extraction method. The most appropriate extraction method should be chosen based on the specific circumstances.

**TABLE 2:** Comparison of different extraction processes for GVO.

Extraction method	Extraction rate	Advantages and disadvantages
Steam distillation	0.3%-0.5%	Advantages: Suitable for samples with high volatile metabolite content, such as plant essential oils. Disadvantages: The ratio of sample to water mixture must be well controlled, otherwise it will affect the separation effect.
Reflux extraction	0.1%-0.3%	Advantages: High efficiency. The reflux extraction method requires less reference solvent, is relatively simple to operate, and has higher efficiency. Disadvantages: High solvent consumption.
Ultrasonic extraction technology	0.05%	Advantages: The extraction efficiency is high, and the temperature during the time is low. Disadvantages: Issues such as high container requirements, noise, and equipment amplification.
Supercritical fluid extraction	1.12%	Advantages: High extraction efficiency, recyclable extraction fluid, preventing pollution to the human body and environment during the extraction process. Disadvantages: The recovery rate is influenced by the matrix in the sample; Extracting polar substances requires adding polar solvents and operating under high pressure, resulting in higher equipment investment.

### 3.1 Traditional extraction methods

#### 3.1.1 solvent extraction (SO)

Solvent extraction is a common method used in practice. Based on the solubility properties of GVO, it can be extracted using the soxhlet extraction method or cold immersion method with organic solvents like petroleum ether (30–60°C), ether, or carbon tetrachloride. The working principle involves the solvent penetrating the cell membrane of botanical drugs, dissolving soluble substances, creating a concentration difference between the inside and outside of the cells, and allowing the solute to permeate out of the cell membrane (Kuang, 2011). After vacuum distillation to eliminate organic solvents, the extract is obtained. Subsequently, hot ethanol is employed to dissolve the extract, which is then cooled, filtered to remove impurities, and the ethanol is reclaimed to obtain clean oil.

The extract can also be re-distilled to acquire a purer essential oil. Studies have explored the extraction of volatile oil using various solvents. Research indicates that the extraction process utilizing water as the solvent can yield the highest levels of phenolic substances and flavonoids (El et al., 2011). This extraction method is straightforward, practical, and enables the extraction of the natural metabolites of plant volatile oil. However, extracting essential oils through leaching with organic solvents is more intricate and often leads to significant solvent residue problems.

#### 3.1.2 Steam distillation method

Research has shown that steam distillation is the most efficient method for obtaining volatile oils, with an extraction efficiency of 93% according to studies (Aziz et al., 2018). The volatile oil is not mixed with water. When the combined vapor pressures of the volatile oil and water equal the atmospheric pressure, the solution boils. If further heated, the volatile oil can be distilled out with water vapor.

During extraction, the crude powder of the raw material can be soaked in water in a still and then directly heated and distilled, or the raw material can be placed on a perforated partition plate net. As the steam generated by heating the water passes through the raw material, the volatile oil is heated and distilled out simultaneously with the water vapor. Collect distillate, cool it and separate the oil layer (Pei, 2016). This method for extracting GVO offers advantages such as simple equipment, easy operation, low cost, large yield, and high recovery rate of volatile oil, However, it should be noted that the raw materials are prone to coking due to the intense heat. Additionally, the heating of volatile oil during the extraction process can lead to chemical reactions such as molecular isomerization, which can affect the composition and reduce the value of the volatile oil (Ma et al., 1985).

Although traditional extraction methods are commonly used in production, they come with some inherent drawbacks. Apart from long extraction times, they necessitate a large amount of solvent and energy. Prolonged contact with hot water or steam can degrade certain metabolites and hydrolyze them. Furthermore, the lack of adjustable parameters in these methods makes it challenging to control the process selectivity and essential oil concentration (Yang et al., 2014).

### 3.2 Modern extraction methods

#### 3.2.1 Supercritical fluid extraction (SFE)

For the extraction of plant volatile oils, supercritical fluid extraction (SFE) is a relatively new and efficient method. SFE is faster, more convenient, and more selective than traditional extraction methods, with higher extraction rates and lower temperatures. In a study, the process of extracting volatile oil from supercritical CO_2_ was optimized by using raw sun-dried *P. ginseng* as raw material. Response surface analysis was employed to determine the optimal extraction conditions (Cui et al., 2016). The results indicated that an extraction pressure of 38 MPa, an extraction temperature of 55°C, a static extraction time of 2 h, and a dynamic extraction time of 1 h resulted in an extraction rate of 1.12%. This method allows for the simultaneous separation of high and low boiling point substances, resulting in a product that is richer in oil metabolites. In addition, it enables the extraction of both volatile and non-volatile GVO, significantly improving the overall yield (Pourmortazavi and Hajimirsadeghi et al., 2007). In the study of *P. ginseng* seed oil extraction, it was found that supercritical fluid extraction yielded higher oil content compared to compression or solvent extraction. The highest yield of *P. ginseng* seed oil extracted by supercritical fluid extraction was 17.48% at 500 bar and 65°C (Lee et al., 2013). This technology utilizes CO_2_ as a supercritical fluid, which prevents the destruction of active metabolites and facilitates the development of new drugs. Furthermore, it reduces labor requirements and the use of organic solvents, thereby reducing pollution from the three wastes, making it a modern technology for the extraction of natural essential oils that is vigorously promoted and widely used.

#### 3.2.2 Microwave-assisted extraction method

Microwave-assisted water distillation (MAHD), which employs water as a solvent, is a sustainable and eco-friendly approach for extracting volatile oils from plants (Golmakani and Rezaei, 2008). During the extraction process, microwave power, liquid-material ratio, extraction time and other parameters have a significant impact on the extraction efficiency. Compared with traditional extraction methods, MAHD significantly shortens extraction time and improves extraction efficiency of essential oils (Shang et al., 2020). There are studies using this method to extract essential oil and polyphenols from camphor leaves, and the yield of essential oil under optimal conditions is 3.26% ± 0.05%. Microwave radiation has the potential to harm cell membranes through cell expansion, modification of intracellular structures, impairment of oil-rich glands and cells, acceleration of the movement of aqueous solutions, and dispersion of internal metabolites (Chen et al., 2016).

#### 3.2.3 Ultrasound-assisted extraction (UAE)

The ultrasonic extraction method is the use of ultrasound cavitation, mechanical effects, and thermal effects to increase the frequency and speed of the molecular movement of substances, to promote contact between the solution and the material, from the target to obtain more metabolites (Raj and Dash, 2020; Yang et al., 2021). It has the advantages of time-saving, energy-saving, and low-temperature extraction is conducive to the protection of active metabolites, it is a rapid and efficient new extraction method.

In one study, raw natural-dried *P. ginseng* powder was used as raw material and ether as solvent in a soxhlet extractor with ultrasonic cleaner at reflux for 90 min in this method. The ether was recovered to obtain the ether leachate, which was subjected to hydrodistillation to collect the distillate. Extracted with ether 5 times, followed by dehydration with anhydrous sodium sulfate and drying to a constant weight. The content determination results revealed that the volatile oil content obtained from the 90-min extraction using the ultrasonic extraction method was in line with the findings reported in the literature (Song, 1991). In another study, ultrasound-assisted pretreatment extraction (UAPE) was employed to extract essential oils from the peels of Tribute citrus (TC) peels, resulting in significantly higher yields compared to traditional hydrodistillation (HD). It has been demonstrated that ultrasonic extraction has a higher extraction rate compared to the conventional method. However, this technique is not suitable for large-scale production (Li et al., 2022).

## 4 The pharmacological effects of GVO and its mechanism of action

Recently, the pharmacological properties of *P. ginseng* have been discovered, revealing its potential in areas such as anti-aging, anti-diabetes, anti-cancer, analgesia, antipyretic, anti-stress, anti-fatigue, sedation, and protein-promoting activities (Zhou et al., 2023). The study mainly focuses on the elaboration of ginsenosides, *P. ginseng* polysaccharides. At present, it has been discovered that the fat-soluble metabolites of *P. ginseng* possess anti-inflammatory, antitussive, antihypertensive, anti-fatigue, anti-tumor, cholesterol-lowering, and central-nervous-exciting effects. In addition, it is worth mentioning that the pharmacological effects and chemical composition of *P. ginseng* can be influenced by various factors, including species, geographic location, cultivation, environment, harvesting, storage, and post-harvest processing.

### 4.1 Cardiovascular effects

GVO has a beneficial therapeutic effect on cardiovascular diseases. The petroleum ether extract of *P. ginseng* has been shown to significantly inhibit diacylglycerol acyltransferase (DGAT) (Lee et al., 2004) and acyl-CoA: cholesterol acyltransferase (ACAT) (Rho et al., 2005) in rat liver microsomes. ACAT has been explored as a potential target for drug intervention in hyperlipidemia and atherosclerosis (Chhabria and Mahajan, 2009). The preventive mechanism involves inhibiting ACAT in the intestines, liver, and arteries, thereby reducing plasma total cholesterol and low-density lipoprotein cholesterol levels, preventing cholesterol esterification, and reducing cholesterol deposition in arterial walls (Kwon et al., 1997). Spectral analysis identified the chemical structures of metabolites in the petroleum ether extract as (9*R*,10*S*)-epoxy-16-heptadecene-4, 6-diyne-3-one, (9*R*,10*S*)-epoxyheptadecan-4,6-diyne-3-one and 1-methoxy-(9*R*,10*S*)-epoxyheptadecan-4,6-diyne-3-one, which inhibit ACAT activity in a dose-dependent manner with IC_50_ values of 35 µM, 47 µM, and 21 µM, respectively. Additionally, ACAT inhibitors isolated from the hairy roots of *P. ginseng*, identified as panaxynol, panaxydol, panaxydiol, and panaxytriol, inhibit rat liver ACAT with IC_50_ values of 94, 80, 45, and 79μM, respectively.

In myocardial ischemia, panaxynol reduces ST-segment elevation by decreasing serum MDA and CTn-I levels, increasing SOD, GSH, and GSH-Px enzyme activities, and enhancing NO concentration and NOS activity, thereby mitigating oxidative damage and myocardial injury (Alanko et al., 1994). GVO has been less well studied in cardiovascular disease, and limited data are available for reference. However, the pharmacological effects of panaxynol in ameliorating myocardial injury have also been demonstrated in studies in other plants. Inflammatory vesicle protein complex (NLRP3) is involved in innate immunity in ischemic heart disease (Wang et al., 2020). Panaxynol also inhibits the NLRP3 inflammasome via the HMGB1/TLR4/NF-κB axis, significantly reducing myocardial infarction area and apoptosis, and alleviating myocardial damage and neutrophil infiltration (Ding et al., 2023).

### 4.2 Anti-inflammatory effect

Relevant pharmacological studies have proved that GVO has anti-inflammatory effects, as shown in Table 3. The main anti-inflammatory metabolite, panaxynol, can non-competitively inhibit 15-hydroxyprostaglandin dehydrogenase in the cytoplasm (Fujimoto et al., 1998). Zuo Xu conducted a study using xylene to induce mouse ear swelling. After treatment with GVO, the earpieces of different experimental groups of mice were weighed and their swelling and inhibition rates were calculated. The results indicated a significant inhibitory effect of GVO on ear swelling in mice. *In vitro* inflammatory cell experiments showed that GVO possesses the ability to suppress the expression of MyD88 and TLR4 proteins, decrease the phosphorylation level of P65 in RAW264.7 cells, and inhibit the NF-kB signaling pathway, thereby exerting anti-inflammatory effects (Zuo, 2021). Acute lung injury, an acute inflammatory disease, can also be ameliorated by panaxydol (PX), a metabolite of GVO. PX has been shown to significantly improve pathological changes in the lungs of mice, reduce pulmonary edema, inflammation, and ferroptosis (Li et al., 2021). The mechanism involves the selective inhibition and upregulation of the Keap1-Nrf2/HO-1 pathway, which markedly attenuates LPS-induced inflammation and ferroptosis.

**TABLE 3:** The targets and mechanisms of GVO in anti-inflammatory.

Activities	Model	Treatment	Mechanism	References
GVO	Balb/c mice RAW264.7 Cells	Mouse:10%(v/v), 5%(v/v), 1%(v/v) Cells:1 μg/mL, 0.8 μg/mL, 0.6 μg/mL	NF-KB pathway	Zuo (2021)
Panaxydol	C57BL/6 mice BEAS-2B cells	C57BL/6 mice:20 mg/kg panaxydol BEAS-2B cells: 10, 20, 40, and 80 μg/mL	Keap1-Nrf2/HO-1 pathway	Li et al. (2021)
Chang Shen Hua volatile oil	Male SD rats PC12 cells	Male SD rats:0.1 mL/kg, 0.05 mL/kg PC12 cells : 80 μg/mL, 40 μg/mL, 20 μg/mL	cAMP-PKA-CREB signaling pathway	Shuangli et al. (2024)

Depression, often linked to inflammatory factors, is another condition where GVO shows promise. Depressed patients typically exhibit elevated levels of cytokines such as IL-1β and TNF-α (Rethorst et al., 2013). A novel herbal inhalation preparation combining GVO with other essential oils, known as CSHVO, has been developed. CSHVO has been shown to enhance the proliferation and viability of PC12 cells by inhibiting cort-induced apoptosis. Additionally, CSHVO intervention significantly reduced the expression levels of pro-inflammatory cytokines IL-1β, IL-6, TNF-α, and IFN-γ, while increasing the levels of anti-inflammatory cytokines IL-4 and IL-10. This suggests that the anti-depressive activity of CSHVO may be related to the inhibition of inflammatory cytokine release and the alleviation of neuroinflammation (Shuangli et al., 2024). Moreover, panaxynol from other plants has been shown to exert anti-inflammatory effects by inhibiting the secretion of inflammatory cytokines TNF and IL-6 in BV-2 microglial cells, preventing their overactivation. This is achieved through the suppression of the NF-κB/i-κB-α inflammatory signaling pathway, which increases the secretion of brain-derived neurotrophic factor (BDNF) and tyrosine kinase receptor B (TrkB) proteins in the hippocampus of mice, thereby achieving anti-depressive effects (Zhao et al., 2021).

In summary, the anti-inflammatory effects of GVO are mediated through multiple mechanisms, including the inhibition of key inflammatory pathways and cytokines, as well as the modulation of neuroinflammatory responses. These findings highlight the potential of GVO as a therapeutic agent for inflammatory diseases and related conditions. Panaxydol in *P. ginseng* volatile oil has anti-inflammatory effects. Panaxynol from other plants has shown anti-inflammatory effects, and it remains to be investigated whether it has the same effect in GVO.

### 4.3 Antibacterial effects

Pharmacological studies have shown that the antibacterial mechanism of GVO may involve the synergistic action of multiple metabolites, such as disrupting bacterial cell wall and membrane permeability, affecting bacterial energy metabolism, and inhibiting protein and nucleic acid synthesis (Chen and Fan, 2016). The volatile oil present in the outer cork layer and phloem of *P. ginseng* roots has inhibitory effects on the growth of various Gram-positive bacteria, including *Staphylococcus*, *Streptococcus*, *Diphtheria*, *Listeria*, and *Streptococcus.*

Panaxynol exhibits good antibacterial activity against *Staphylococcus aureus*, *Mycobacterium tuberculosis*, *Bacillus subtilis*, *Gram-negative bacteria*, and *Escherichia coli* (Bae et al., 2001). Gram-negative bacteria are known to cause inflammation in the lungs, and lipopolysaccharide (LPS), the principal metabolite of the outer membrane of gram-negative bacteria, is considered one of the major causes of lung diseases (Kolomaznik et al., 2017). Panaxydol can inhibit the activity of LPS, thereby effectively inhibiting gram-negative bacteria. *Helicobacter pylori* (HP) is recognized a risk factor for gastric cancer and plays a crucial part in the development of gastritis and peptic ulcers (Warren and Marshall, 1983). Pathogenic HP produces urease, an enzyme that breaks down urea into ammonia and carbamate. Research has confirmed that panaxytriol can achieve anti-Helicobacter pylori effects by inhibiting gastric urease and gastric *H+/K +* ATPase (Bae et al., 2001) (Table 4).

**TABLE 4:** The targets and mechanisms of GVO in antibacterial.

Activities	Disease	Model	Treatment	Mechanism	References
Panaxytriol	*Helicobacter pylori* (HP)	HP ATCC43504 strain	panaxytriol: 50 μg/mL	Inhibition	Bae et al. (2001)
Hydrophobic fraction of red ginseng ethanol extracts (Panaxynol and panaxydol)	Acne	*Propionibacterium acnes* female volunteers. men and women age 19 to 40 years	12.5、6.25 and 3.12 mg/mL 3 mg/g of RGEF	Inhibition	Contassot and French (2014)
Panaxytriol	*Helicobacter pylori*	H.Pylori Hela cells	*H.Pylori* (IC50: 0.05, 0.046 mg/mL) Hela cells: 6 mg/mL	Inhibition	Kim et al. (2003)

Moreover, polyacetylene also has good antifungal (Xie et al., 2022) and antibacterial effects (Kim et al., 2003). Acne is a chronic inflammatory skin disease caused by excessive sebum secretion, proliferation of *Propionibacterium acnes*, and inflammatory response (Contassot and French, 2014). Panaxynol and panaxydol have shown selective inhibitory effects on *P. acnes,* making them effective in treating acne (Hou et al., 2019).

### 4.4 Anticancer effects

**FIGURE 9:**
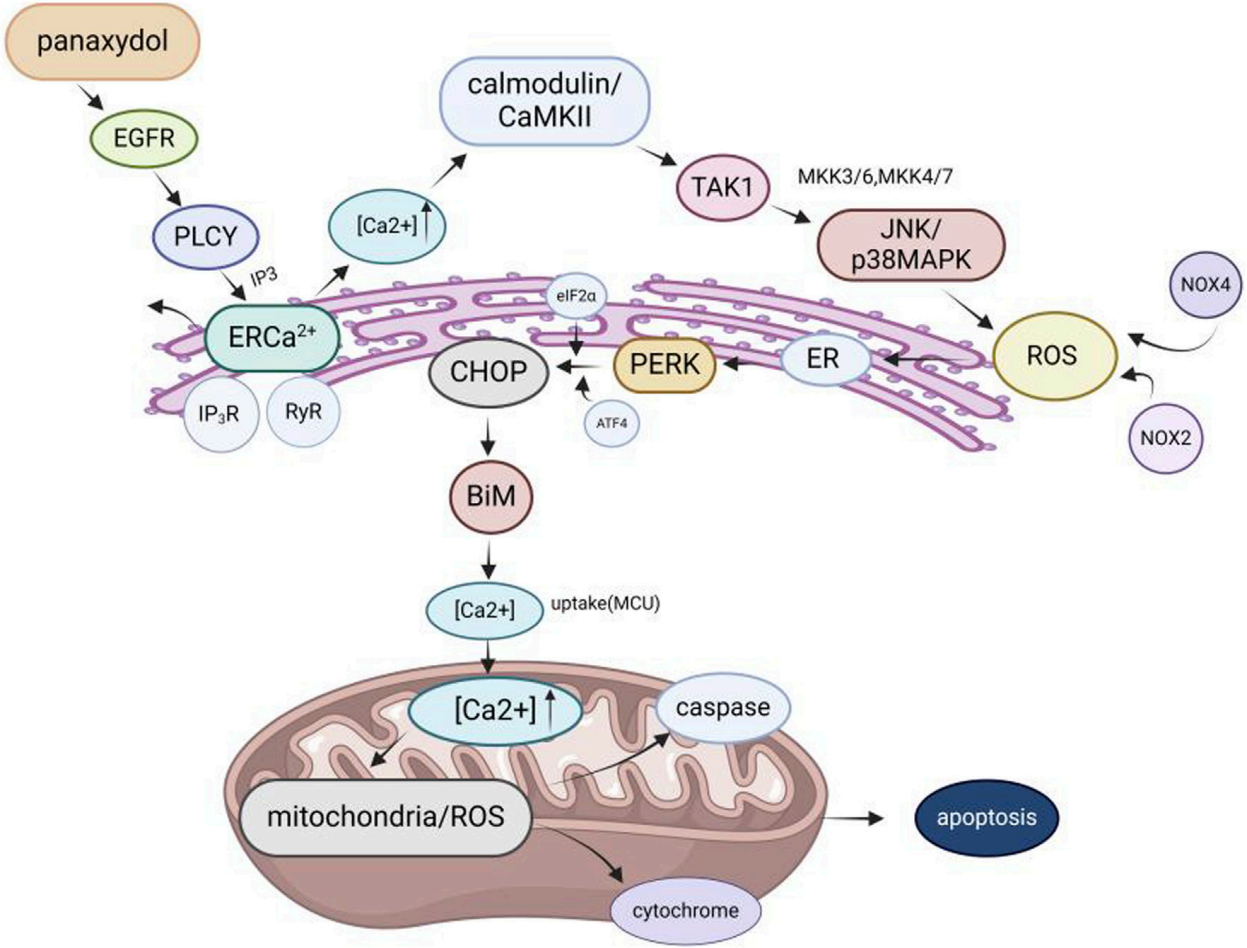
Molecular mechanism of GVO for anti-cancer. The mechanism of panaxydol induced cell apoptosis involves a rapid increase in cytoplasmic Ca2+concentration, with excess Ca^2+^ transferring from the endoplasmic reticulum (ER) to mitochondria. The release of E [Ca^2+^] and the resulting increase in Ca^2+^ activate p38 and JNK, while p38/JNK further activates NADPH oxidase. NADPH oxidase activates and induces oxidative stress, triggering mitochondria-dependent apoptosis.

Among the pharmacological effects of GVO, its anticancer activity has been extensively studied. The anticancer activity of *P. ginseng* is primarily attributed to its lipophilic metabolites. Hexane extraction maximizes the recovery of anticancer active metabolites, demonstrating strong *in vitro* inhibitory effects on the proliferation of human liver and breast cancer cells in a concentration-dependent manner (Lee et al., 2009). Polyacetylenes such as panaxynol, panaxydol, and panaxytriol are considered the main anticancer metabolites, exhibiting antiproliferative effects on mouse sarcoma, leukemia, human colon cancer, and human ileocecal adenocarcinoma cell lines. Panaxydol accelerates cell cycle progression from G1 to S phase by reducing Cdk1 activity and upregulating p27KIP1 protein expression, inhibiting human renal cell carcinoma proliferation (Sohn et al., 1998; Moon et al., 2000). Panaxydol induces cancer cell apoptosis by inhibiting EGFR activation and endoplasmic reticulum stress, suppressing tumor growth in mice (Figure 9) (Kim et al., 2011; Kim et al., 2016). Panaxynol significantly reduces MMP-2 mRNA and protein levels in melanoma cells (B16F10) at a concentration of 3 μg/mL, inhibiting cancer cell invasion and migration (Yun et al., 2015) (Table 5).

**TABLE 5:** The targets and mechanisms of GVO in anticancer.

Activities	Disease	Model	Treatment	Mechanism	References
Hexane extract of ginseng marc (HEGM), hexane-soluble fraction (denoted as HEG)	Cancer	Human hepatoma (HepG2) and human breast cancer (MCF-7) cell lines	HEG showed strong inhibition of HepG2 (GI50: 21.1 μg/mL) and MCF-7 (GI50: 41.2 μg/mL. HEGM(GI50: 41.7 μg/mL in HepG2 , GI50: 54.4 μg/mL in MCF-7)	Inhibit proliferation	Lee et al. (2009)
Panaxydol	Human malignant tumor cell	Human melanoma cell line SK-MEL-1	5, 10, 20 μg/mL	Inhibit cell cycle	Moon et al. (2000)
Petroleum ether extract of panax ginseng roots	Cancer	Human renal Cells carcinoma (RCC) cell lines (Caki-1, A498, and CURC II)	0, 40, 80 μg/mL	Inhibit cell cycle	Sohn et al. (1998)
Panaxydol	Cancer	BEAS-2B (normal immortalized), 1799 (non-transformed), 1198 (transformed but non-tumorigenic) and 1170-I (tumorigenic) cell lines comprising an *in vivo* lung carci-nogenesis model. Human leukemia T cell line, Jurkat, and a human breast cancer cell line, MCF-7	MCF-7 and Jurkat cells were treated with 50 and 40 μg/mL of panaxydol, BEAS-2B, 1799, 1198 and 1170-I cells were treated with 50 μg/mL panaxydol	Increase in [Ca^2+^]i, activation of JNK and p38 MAPK, and ROS generation through NADPH oxidase and mitochondria	Kim et al. (2011a)
Panaxydol	Cancer	MCF-7 human breast cancer, Pathogen-free female BALB/c or BALB/c nu/nu mice	MCF-7 cells were treated with 20 or 50 μg/mL Mouse: panaxydol (50, 100 mg /kg)	EGFR activation and ER stress mediate panaxydol-induced apoptosis. CAMKII-TAK1-p38/JNK signaling pathway	Kim et al. (2016)
GVO	Gastric cancer	MKN-45 cells, HGC-27 cells	MKN-45 cells: 0 μg/mL、50 μg/mL、100 μg/mL. HGC-27 cells: 150 μg/mL、200 μg/mL and 250 μg/mL	Regulation of EGFR, MAPK1, MAPK3, and IL-6 related signaling pathways	Cui (2022)
Lipid-soluble ginseng extract	Lung cancer	NCI-H460 human lung cancer cell line	0、30、100 μg/mL	Cell cycle arrest and apoptosis induction.	Kang et al. (2011)
Fat-soluble metabolites	Lung cancer	NCI-H1299 human lung cancer cell line	0、50、100、150、200 and 250 μg/mL)	EGFR、KDR、MAPK3、PTPN11 and CTNNB1 signaling pathway	Gao et al. (2023)
Panaxynol	Lung cancer	Human NSCLC cell lines, human lung Epithelium and human retinal pigmental epithelium (RPE)	Human NSCLC cell lines: 5 mmol/L. BEAS-2B: <1 μm, HBE: 1 μm RPE: 1 μm	Inhibition of Hsp90	Le et al. (2018)
Panaxynol	Pancreatic cancer	Panc-1	80 μg/mL	Inhibits the migration and invasion of pancreatic cancer stem cells Attack ability	Wang et al. (2015)
*P.ginseng* seed oil (GSO)	Breast cancer	MCF-7 breast cancer cells	1 μL/mL	Causes apoptosis of ER+ breast cancer cells via PKC activation	Kim et al. (2020)
Panaxydol	Hepatocarcinoma	Human liver carcinoma cell line HepG2	4, 6 and 9 μm	Blocks the cell cycle progression of HepG2 cells	Guo et al. (2009)

In addition, some rare metabolites in the volatile oil of *P. ginseng* also have anticancer activity, such as β*-*elemene, (Peng et al., 2006), d-limonene (Anandakumar et al., 2021), and α-humulene (Chen et al., 2019). Among these metabolites, β-elemene, a sesquiterpene effective active monomer found in *P. ginseng* essential oil, demonstrates significant anticancer activity and is classified as a class II non-cytotoxic antitumor drug in China. It is clinically for the treatment of rectal cancer (Wang et al., 2022), breast cancer (Xie and Wang, 2022). Its anticancer mechanism may induce apoptosis in cancer cells through a variety of pathways, such as the ROS-mediated mitochondrial pathway, cellular oxidative dysfunction, the caspase-dependent mitochondrial death pathway, and inhibition of the PI3K/Akt pathway (Qureshi et al., 2019). Inducing autophagy in cancer cells by targeting multiple molecular targets such as kinases, transcription factors, growth factors, their receptors, and proteins (Zhai et al., 2019).

#### 4.4.1 Anti-gastric cancer

GVO has shown promising anti-cancer properties, particularly against gastric cancer. Key metabolites such as linoleic acid, panaxynol, methyl linoleate, palmitoleic acid, and oleic acid have been identified to interact with critical targets in gastric cancer, including EGFR, MAPK1, MAPK3, and IL-6, significantly inhibiting the proliferation of gastric cancer cells (Cui, 2022). This inhibition is dose- and time-dependent, as demonstrated in vitro experiments with SGC-823 gastric cancer cells, where GVO treatment resulted in a marked reduction in glycogen and succinate dehydrogenase content, and a significant decrease in DNA content after 72 h of administration (Wang et al., 1992). This suggests that the mechanism by which GVO inhibits gastric cancer cell growth may involve disruptions in DNA, carbohydrate, and energy metabolism. Additionally, panaxytriol has been shown to exhibit cytotoxicity against human gastric cancer cell line MK-1, enhancing the cytotoxic effects of MMC by decreasing membrane fluidity and promoting MMC accumulation in MK-1 cells (Matsunaga et al., 1994).

#### 4.4.2 Anti-lung cancer

GVO, particularly its polyacetylenes, has demonstrated significant anti-tumor activity against lung cancer and has a strong inhibitory effect on lung cancer cell lines at high concentrations (100 μg/mL). This anticancer mechanism initiates the activation of caspase-8 and caspase-9, which in turn activates caspase-3 to induce cleavage of PARPs. This process results in cell cycle arrest at the G0/G1 phase, thereby inhibiting lung cancer cell proliferation (Kang et al., 2011). Furthermore, GVO metabolites downregulate the levels of EGFR, KDR, MAPK3, PTPN11, and CTNNB1 proteins in lung cancer cells, affecting the PI3K/Akt and RAS/ERK pathways, which are crucial for cell proliferation and survival proteins regulated by Hsp90 (Gao et al., 2023). Panaxynol treatment also significantly inhibits the interaction between HIF-1α and Hsp90, reducing HIF-1α protein levels and VEGF mRNA levels in a dose-dependent manner, further supporting its anti-lung cancer effects (Lee et al., 2018; Le et al., 2018).

Furthermore, panaxynol has been found to decreases proliferation and self-renewal of pancreatic cancer PANC-1 stem cells. Its principle may inhibit the migratory ability of SW1990 cells by down-regulating the expression of Ki67, PCNA, Vimentin, and MMP-9 as well as up-regulating the expression of E-cadherin, thus exerting its anti-tumor effect (Wang et al., 2015). In addition, some studies also illustrated the phenomenon of panaxynol inhibiting epithelial-mesenchymal cell transformation at the molecular level. It exerts its anti-tumor effects by down-regulating the expression of Vimentin and MMP-9 and up-regulating the expression of E-cadherin. *P. ginseng* seed oil (GSO) in combination with tamoxifen inhibits the growth of ER + breast cancer cells. It induces apoptosis in ER + breast cancer cells by activating PKC (Li et al., 2012), which leads to the breakdown of caspase-9, caspase-3, and PARP (Kim et al., 2020). α-Humulene (Legault et al., 2003), β-sitosterol (Shin et al., 2016) and (*Z*)-β-farnesene (Afoulous et al., 2013) have been shown to be associated with the anticancer activity of several plant essential oils. The fat-soluble metabolites panaxydol and panaxynol can inhibit liver cancer and block the cell cycle progression from G1 phase to S phase at high concentrations, thereby inhibiting the proliferation of human kidney cancer cells (Guo et al., 2009).

**FIGURE 10:**
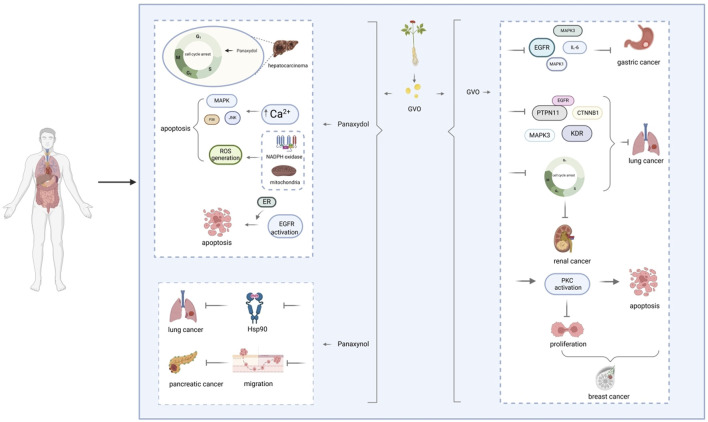
Graphic summary of GVO against cancer.

In conclusion, the anti-cancer effects of GVO are primarily attributed to its ability to interfere with key signaling pathways and cellular processes involved in cancer cell proliferation and survival (Figure 10). The active metabolites of GVO, such as linoleic acid, panaxynol, and panaxytriol, exhibit potent inhibitory effects on gastric and lung cancer cells through mechanisms involving apoptosis induction, cell cycle arrest, and disruption of critical protein interactions. These findings highlight the potential of GVO as a valuable therapeutic agent in the treatment of gastric and lung cancers.

### 4.5 Anti-aging, anti-oxidation

GVO has demonstrated significant anti-aging and antioxidant properties, which are crucial for mitigating the effects of oxidative stress and cellular aging (Alanko et al., 1994). Studies have shown that panaxynol and panaxydol in GVO can prolong the lifespan and health span of model organisms such as *Caenorhabditis elegans*. The underlying mechanisms involve the upregulation of autophagy-related genes such as atg-4.2, atg-7, lgg-2, and cyd-1, as well as the increased expression of superoxide dismutase 1 (sod-1). These genetic modifications enhance the organism’s ability to manage oxidative stress, thereby promoting longevity and health without compromising reproductive capacity. Additionally, GVO has been found to activate SOD activity and autophagy, which are indicative of hormesis—a process where low doses of a stressor can stimulate beneficial effects on the organism (Table 6).

**TABLE 6:** The targets and mechanisms of GVO in antioxidant.

Activities	Disease	Model	Treatment	Mechanism	References
GVO	Extend life	*Caenorhabditis elegans* strains	12.5、25 and 50 μg/mL	Antioxidant, upregulation of autophagy-related genes atg-4.2, atg-7, lgg-2 and cyd-1	Wang et al. (2022)
Panaxydol	Antioxidant	BEAS-2B (normal immortalized), 1799 (non-transformed), 1198 (transformed but non-tumorigenic) and 1170-I (tumorigenic) cell lines	50 μg/mL	[Ca^2+^]i increase, JNK and p38 MAPK activation, and ROS generation through NADPH oxidase and mitochondria.	Nie et al. (2008)

**FIGURE 11:**
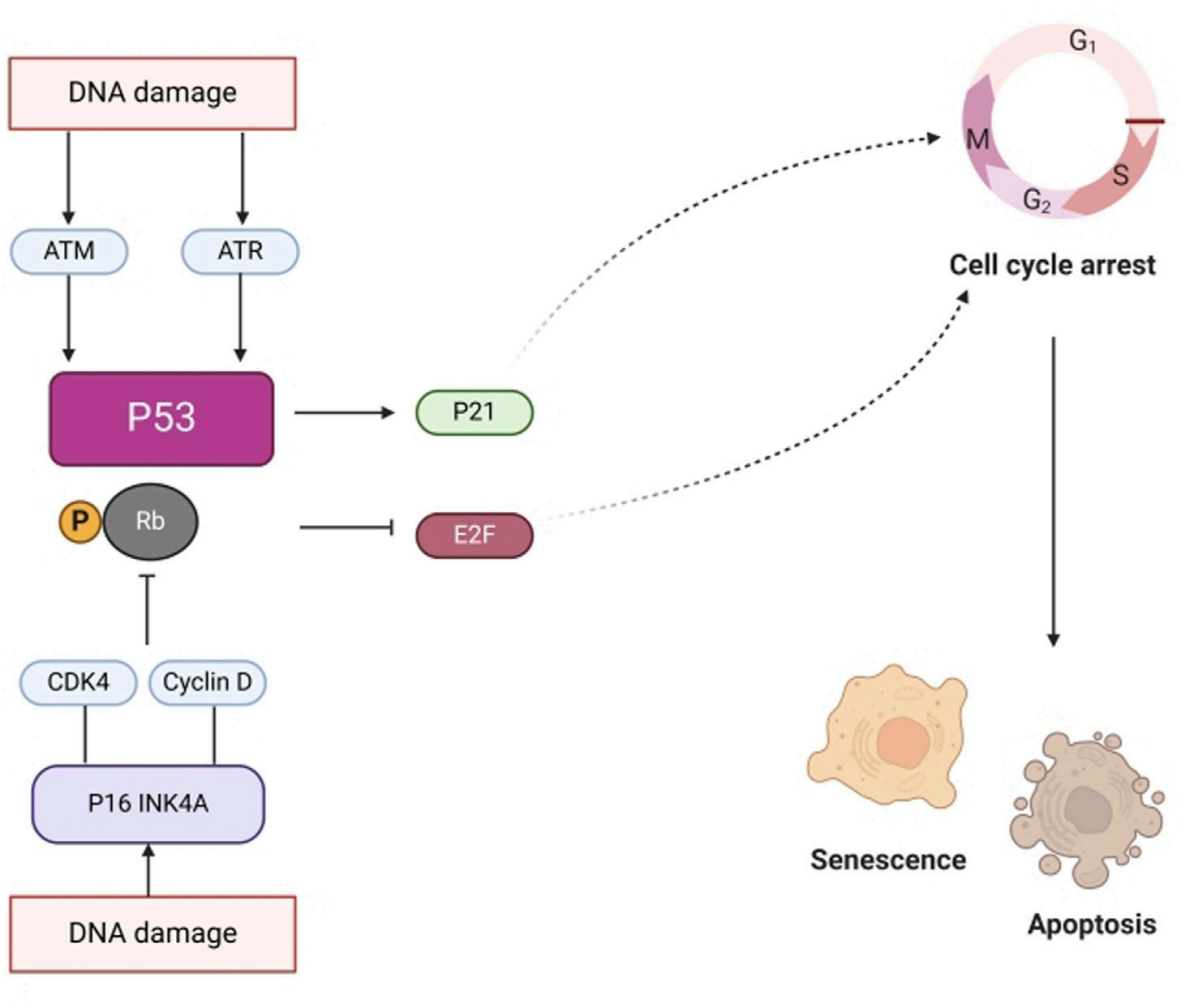
DNA damage-induced senescence pathways.

Senescence is a state in which cell division permanently ceases and cells die. Aging is closely associated with major factors such as DNA damage and mitochondrial dysfunction. Figure 11 illustrates that DNA damage triggers the activation of the ataxia telangiectasia mutated gene (ATM), Rad3 related gene (ATR), p53 pathways (Thompson, 2012), and cell cycle dependent protein kinase inhibitor p21, thereby promoting cell cycle arrest and inducing aging. Moreover, DNA damage activates P16INK4a and inhibits the binding of CDK4 to cyclin D. This prevents phosphorylation of retinoblastoma (RB), leading to inhibition of E2F dependent gene expression and inhibition of G1/S cell cycle progression. GVO can repair damaged pathways by increasing the expression of autophagy substrate p62 protein to delay aging (Su et al., 2023).

During oxidative stress, the increase in reactive oxygen species (ROS) and the decrease in antioxidant defense mechanisms have a broad impact on apoptotic and non-apoptotic cell death (Aruoma et al., 2006; Bak et al., 2012b). The main sources of intracellular ROS include NADPH oxidase and the mitochondrial electron transport chain (ETC.) (Ryter et al., 2007). Oxidative stress occurs when there is an excess production of ROS by mitochondria or NADPH oxidase. Cancer cells are more sensitive to oxidative stress, and scertain anticancer drugs work by inducing ROS production (Pelicano et al., 2003; Valko et al., 2006). Panaxydol induces apoptosis by increasing intracellular calcium level, activation of JNK and p38 MAPK and generating ROS dependent on NADPH oxidase (Kim et al., 2011). In addition, panaxydol (PX), panaxynol from other plants also showed good antioxidant activity by inhibiting oxidative stress through activation of Keap1-Nrf2 signaling pathway, reduction of LPO and its related markers, and activation of NO1 and HO - one genes, which resulted in protection of kidneys from damage (Aruoma et al., 2006; Nie et al., 2008). However, whether the antioxidant capacity of panaxydol in *P. ginseng* is related to the Keap1-Nrf2 system has not yet been confirmed, and it is expected that scholars will further study this.

In summary, the anti-aging and antioxidant effects of GVO are mediated through a combination of genetic regulation, enhancement of autophagy, and activation of key antioxidant pathways. These mechanisms collectively contribute to the mitigation of oxidative stress and the promotion of cellular longevity, making GVO a promising candidate for further research and potential therapeutic applications in age-related diseases.

### 4.6 Antiplatelet clotting

Research on the anti-platelet aggregation effects of GVO is limited, but it has shown better anti-platelet activity than ginsenosides (Kuo et al., 1990). Panaxynol, a metabolite of GVO, exhibits good anti-platelet aggregation effects by inhibiting ATP release and platelet aggregation (Teng et al., 1989). At a concentration of 0.41 μmol/mL, panaxynol inhibits platelet aggregation induced by arachidonic acid, collagen, ADP, and ionophore A23187 in rabbits, significantly inhibiting thromboxane B_2_ formation. Ginsenoside prevents secondary aggregation of ATP induced by adrenaline and ADP, completely blocking ATP release. It also inhibits platelet aggregation induced by collagen, ADP, and thrombin, as well as ATP release and thromboxane formation in platelets (Kwon et al., 1997). The addition of 25 mg (0.0025% of the total diet) of *P. ginseng* lipophilic fraction to the diet can be antithrombotic (Park et al., 1996). After feeding them to rats stimulated by thrombus and collagen, cGMP levels were significantly higher than those of rats given only 15% corn oil. In addition, by adding LF to thrombin- and collagen-stimulated platelets, cGMP and CAMP levels are elevated. This indicates that LF has a direct promoting effect on cGMP. It can be seen that the addition of LF to the diet regulates cGMP and CAMP levels, resulting in the inhibition of thrombin- or collagen-induced platelet aggregation in rats.

### 4.7 Nutrition and protection of nerve cells

Panaxydol (PND) and panaxynol (PNN) protect cortical neurons from toxicity-induced damage by reducing the upregulation of pro-apoptotic gene Bax and the downregulation of anti-apoptotic gene Bcl-2 at a concentration of 5 µM, suggesting potential benefits in reducing neurodegeneration in Alzheimer’s disease (Nie et al., 2006). In a mouse maze experiment, panaxynol and acetylenic triol at a dose of 20 mg/kg/day for three consecutive days improved scopolamine-induced memory deficits. At concentrations greater than 2mM, they significantly affect the neurodevelopment of secondary neurons such as PC12 h and Neuro2a (Yamazaki et al., 2001) (Table 7).

**TABLE 7:** The targets and mechanisms of GVO in neuroprotection.

Activities	Disease	Model	Treatment	Mechanism	References
Panaxydol and panaxynol	Apoptosis in cortical neurons	Pure cortical neurons	5 μm	Downregulation of the pro-apoptotic gene Bax and downregulation of the anti-apoptotic gene Bcl-2 were reduced	Guo et al. (2021)
Panaxynol and the acetylenic triol	Memory impairment	PC12h cells and Neuro2a cells ddY strain mice	>2 μm	Affects the neuritogenesis of paraneurons like PC12h and Neuro2a,	Yamazaki et al. (2001)
Panax ginseng essential oil and Acorus tatarinowii essential oil, Albizia julibrissin flower essential oil	Depression	PC12 cells Male SD rats	PC12 cells: 20、40、80 μg/mL Male SD rats: 0.1 mL/kg、 0.05 mL/kg	cAMP-PKA-CREB pathway	Shuangli et al. (2024)

Clinical and animal studies indicate that depression, a common central nervous system disorder, is associated with reduced or deficient serotonin (5-HT) (Yatham et al., 2000; Ferrari and Villa, 2017) and dopamine (DA) (Sykora et al., 2013). The combination of *P. ginseng* essential oil, Acorus tatarinowii essential oil, and Albizia julibrissin flower essential oil (CSHVO) from the classic Chinese herbal prescription Kai Xin San (KXS) has shown efficacy in improving depression (Cao et al., 2023). CSHVO significantly increases monoamine neurotransmitter levels in the brain tissue of depressed rats and improves hippocampal pathology. By regulating the cAMP-PKA-CREB signaling pathway, CSHVO promotes neuronal development, repairs nerves, and exerts antidepressant effects (Zhang et al., 2024). Panaxynol from other plant species also exhibits neuroprotective effects by inhibiting calcium influx and promoting free radical production, counteracting amyloid-beta 25–35 fragment-induced early neuronal degeneration (Sun et al., 2020). Panaxydol promotes neurite outgrowth in PC12 cells, protecting neurons from neurodegenerative diseases such as Alzheimer’s disease (Li et al., 2018). Panaxydol enhances the expression and secretion of nerve growth factor (NGF) and brain-derived neurotrophic factor (BDNF) in Schwann cells (SCs), improving SC viability and biological characteristics, effectively protecting neurons from degenerative disease damage (He et al., 2009). While panaxydol and panaxynol in GVO also exhibit neuroprotective effects, further research is needed to explore the underlying mechanisms and potential clinical applications as early treatment candidates for Alzheimer’s disease (Zhu et al., 2008).

### 4.8 Other pharmacological effects

In addition to the main pharmacological effects such as anti-inflammatory, antioxidant, and anti-cancer, GVO also has the functions of weight loss (Kim et al., 2011), hair growth, protection against ultraviolet radiation, and reproductive system protection (Mhaibes et al., 2023). Linoleic acid (LA) or beta-sitosterol (SITOS) in volatile oils can stimulate the transition of hair follicles from the resting phase to the early/mid-growth phase. This leads to an increase in follicle density and diameter, as well as the emergence of the hair shaft from the epidermis. These metabolites synergistically induce the expression of β-catenin, phosphorylated glycogen synthase kinase 3β, cyclin D1, cyclin E, and Bcl-2, all of which are associated with hair growth. Furthermore, they activate the Wnt/β-catenin and Shh/Gli pathways, promoting hair follicle development and regeneration.

GVO contains various metabolites with unsaturated double bonds, among which n-hexadecanoic acid is particularly effective as an “anti-mosquito agent” (Jiang et al., 2014). In addition, the volatile oil derived from *P. ginseng* root possesses pharmacological properties that benefit the skin. Through skin friction, it enhances blood circulation and the development of skin cells, while providing protection against cold and ultraviolet radiation.

Cholesterol acyltransferase (ACAT) plays a crucial role in cholesterol uptake, storage, and production and has been explored as a potential target for pharmacological intervention in hyperlipidemia and atherosclerotic diseases (Giovannoni et al., 2003). Polyacetylene metabolites derived from *P. ginseng* root exhibit modest inhibition of ACAT enzymes in rat liver microsomes (Rho et al., 2005). In the MAPK signaling pathway, JNK and p38 are the main mediators of apoptosis in proximal tubule cells (Guo et al., 2021). Panaxynol can inhibit PGDH activity in gastric mucosa, inhibit apoptosis by down-regulating cisplatin and promote the phosphorylation of JNK and p38 in cells and the expression of cleaved caspase-3, thereby improving kidney injury (Fujimoto et al., 1998; Lee et al., 2019). In addition, panaxydol exhibits anti-fatigue properties, which can significantly reduce the levels of oxidative stress markers such as serum LDH, superoxide dismutase and malondialdehyde in forced swimming rats, and inhibit oxidative stress (Shin et al., 2019).

## 5 Discussion

*P. ginseng* is a widely recognized medicinal plant that has a positive effect on immune regulation and the circulatory system (Shergis et al., 2013). It has been extensively utilized in medicine, food and other fields, and has shown promising application prospects (Qin et al., 2018). Ginsenosides are known to have favorable therapeutic effects in cardiovascular diseases (Fan et al., 2020), neurodegenerative diseases (Zarneshan et al., 2022), cancer (Shah et al., 2023), and diabetes (Zhou et al., 2019). *P. ginseng* polysaccharides exhibit anti-inflammatory (Qi et al., 2023), anti-tumor (Li et al., 2014), antioxidant (Xiong et al., 2019), anti-asthmatic, hepatoprotective, anti-depressant, anti-radiation and blood lipid regulating properties (Zhao et al., 2019). Although *P. ginseng* is widely consumed and utilized, there still exists potential investment opportunities in the field of GVO. The current research mainly focuses on the main metabolites of *P. ginseng*, ginsenosides and *P. ginseng* polysaccharides, but there are fewer studies on *P. ginseng* volatile oils. Therefore, this paper summarizes the research results of GVO in recent years and finds that the current research has limitations. For example, in terms of pharmacological effects, in-depth research has been conducted mainly in the direction of anti-cancer. Therefore, this paper summarizes the results of the current study and serves as a starting point for future in-depth studies on GVO.

The volatile oil of *P. ginseng* is a complex mixture composed of hundreds of metabolites, mainly terpenes, including monoterpenes and sesquiterpenes, as well as small aliphatic metabolites and small aromatic metabolites. *P. ginseng* contains two highly abundant polyacetylenes, namely, panaxynol and panaxydol, which are the main metabolites of *P. ginseng* essential oils. Polyacetylene extracted from *P. ginseng* exhibits significant biological effects, including induction of cytotoxicity (Yeo et al., 2017), inhibition of tumor cell proliferation (Kim et al., 2002), inhibition of platelet coagulation function (Teng et al., 1989) and inhibition of diacylglycerol acyltransferase (DGAT) enzyme activity (Lee et al., 2004). The presence of a triple bond in polyacetylene increases its reactivity towards biomolecules (He et al., 2014). In addition, polyacetylene has also been shown to be cytotoxic to many solid and leukemic cell lines (Kim et al., 2002), as well as the ability to enhance the cytotoxicity of other anticancer drugs (Matsunaga et al., 1990; Matsunaga et al., 1994). Moreover, analogs and derivatives of polyacetylene have shown promising anti-inflammatory activity, nutritive neurological effects (Wang et al., 2006), immune enhancement (Chou et al., 2011), anticancer properties (Cheung et al., 2019) and the ability to attenuate the toxicity of a range of anticancer drugs. Thus GVO might be developed as an immune-boosting product or an adjuvant anti-cancer drug.

## 6 Conclusion and future perspectives

As a traditional Chinese medicine, *P. ginseng* has been highly regarded for its therapeutic efficacy. This paper expands our understanding of *P. ginseng* and reveals the complex and diverse chemical compositions and pharmacological activities of GVO. The compositions of the volatile oils from different parts of the *P. ginseng* plant, their contents, extraction methods and pharmacological activities, as well as the mechanisms of action of their major molecules, were systematically summarized. Several factors affect the composition of volatile oils: growth year, collection season, geographical region, extraction method, extraction site, etc. The composition of the volatile oil in turn affects its biological activity. Modern techniques such as supercritical fluid extraction, microwave-assisted extraction and ultrasound-assisted extraction offer promising ways to improve the yield and bioavailability of GVO. Therefore, standardization of these extraction parameters is essential to ensure the consistency of volatile oil quality and efficacy.

From an expert’s point of view, the development and utilization of GVO is promising but faces significant challenges. For example, there are many metabolites of *P. ginseng* volatile oil that are not widely recognized, so there is no international standardized nomenclature. As a result, the metabolite names do not correspond to each other when the data are summarized, so the composition summary of GVO is not complete. The pharmacological activities of GVO, including cardiovascular, anti-inflammatory, antimicrobial, anticancer, anti-aging, and neuroprotective effects, highlight its potential as a multifaceted therapeutic agent. However, the low content of volatile oil in *P. ginseng*, coupled with its lipophilicity, degradability and volatility, poses a significant barrier to its clinical application and efficacy. Although, the pharmacological mechanisms of *P. ginseng* volatile oil metabolites (especially panaxynol) highlight the potential for new drug development, little is known about their pharmacokinetic profile. Therefore, in-depth pharmacokinetic studies of GVO should be performed to check for the presence of active metabolites. The identification of these metabolites may provide key information on the bioactive forms of GVO and its pharmacological mechanisms. Therefore, this area may become a new focus for future research. The current literature on GVO remains limited and quality assessment studies are incomplete. This gap highlights the need for more comprehensive and rigorous studies to fully elucidate the therapeutic potential and safety of GVO.

In conclusion, although GVO has a wide range of pharmacological activities, it has been less studied in the areas of immunization, antimicrobial and antiplatelet coagulation. This necessitates further research to address the existing challenges and optimize its clinical applications. Future studies should focus on improving extraction techniques, standardizing quality assessment, and exploring synergistic or antagonistic effects of volatile oil metabolites. Only in this way can we fully utilize the potential of GVO as a valuable resource in the fields of medicine and healthcare.
