# Supplementary material for: Phytochemistry, pharmacological effects and mechanism of action of volatile oil from Panax ginseng C.A.Mey: a review
Source: Front Pharmacol. 2024 Aug 13;15:1436624. doi: 10.3389/fphar.2024.1436624 (PMC11347760; doi:10.3389/fphar.2024.1436624)
Supplement: Supplementary file 1 [file DataSheet2.PDF]

frontiers

Supplementary Material

1.1 Supplementary Figures

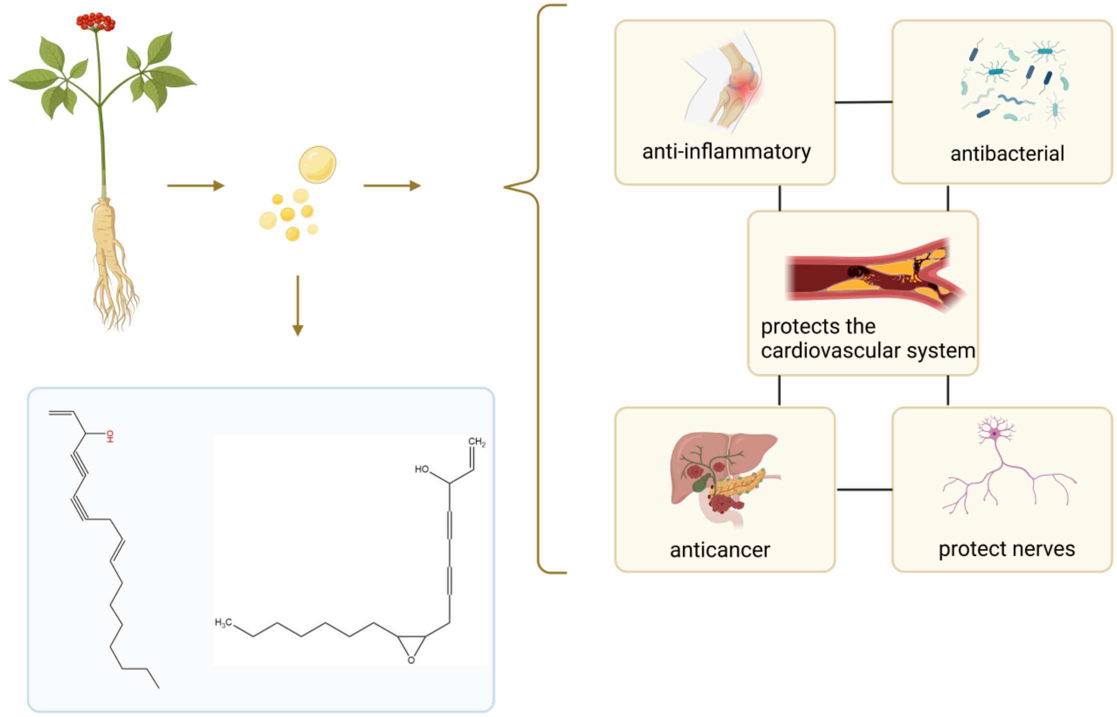

Figure 1. Summary of the composition and effect of GVO. GVO has a special aroma, and its main metabolites are linoleic acid, panaxynol, panaxydol and so on. It has anti-inflammatory, antioxidant, anti-aging, anti-cancer, neuroprotective, and fatigue-relieving properties.

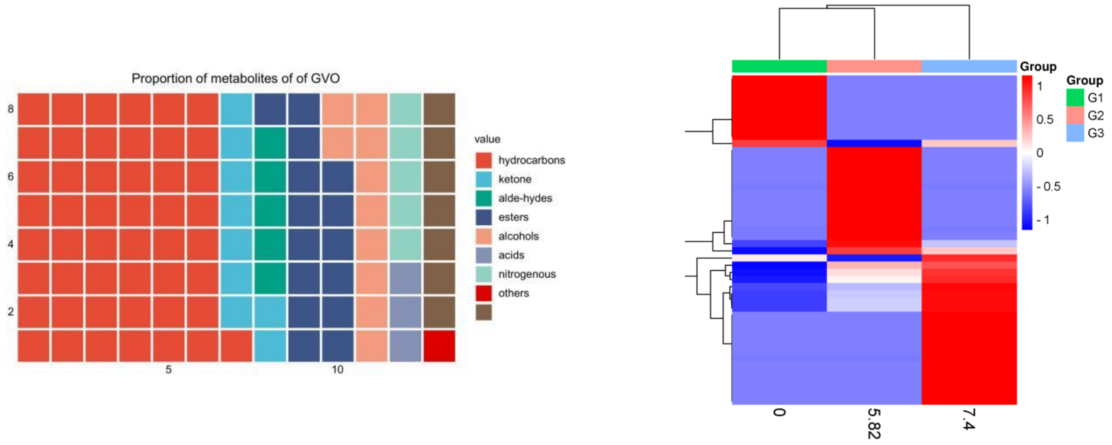

Figure 2. A. Proportion of different types of metabolites of GVO. The chemical composition of GVO is relatively complex, in which hydrocarbon metabolites accounted for 49.7%, ketone metabolites for 9.5%, aldehyde metabolites for 5.4%, ester metabolites for 15%, alcohol metabolites for 10%, acid metabolites for 3.2%, nitrogenous metabolites metabolites for 5.9%, and other metabolites contained 14%. **B.** Heat map of chemical composition differences among fresh ginseng G1, white ginseng G2, and red ginseng G3. The chemical composition of G1, G2 and G3 of fresh ginseng, G2 and G3 showed that there were differences between G3 and G1 and G2, and the differences between G1 and G2 were small.

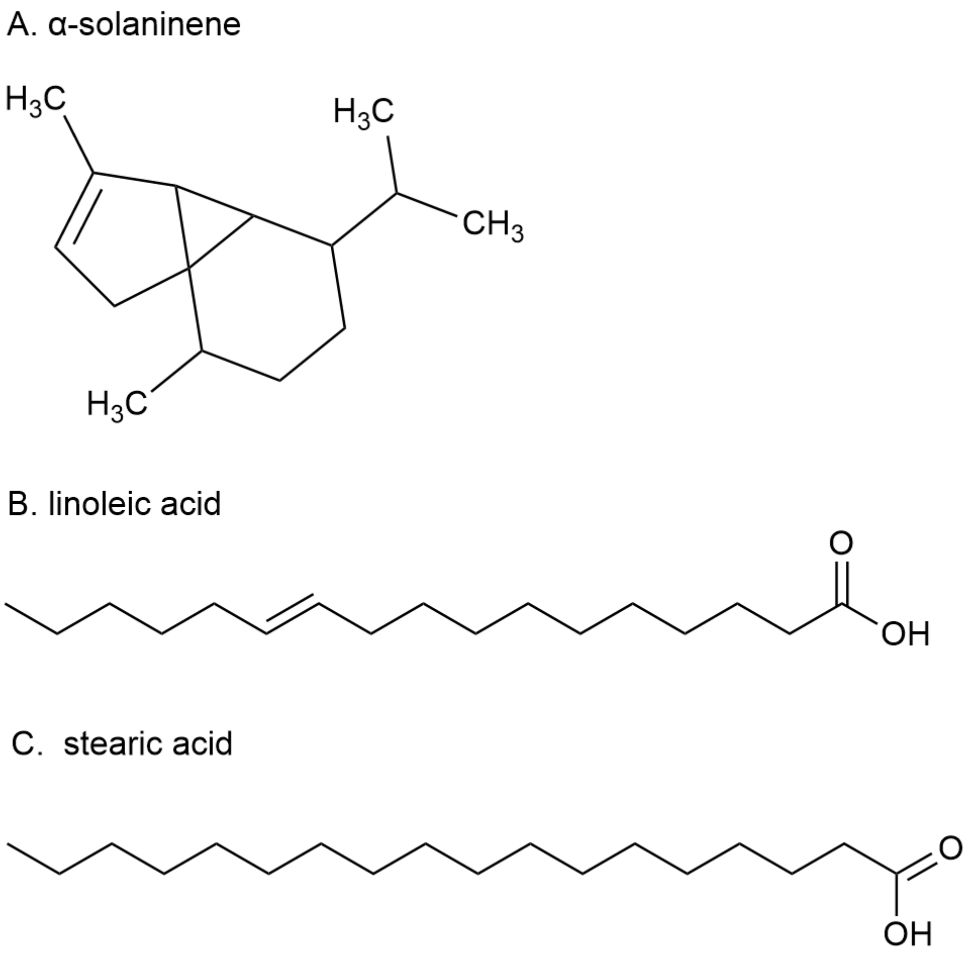

Figure 3. The main metabolites of GVO in flower buds.

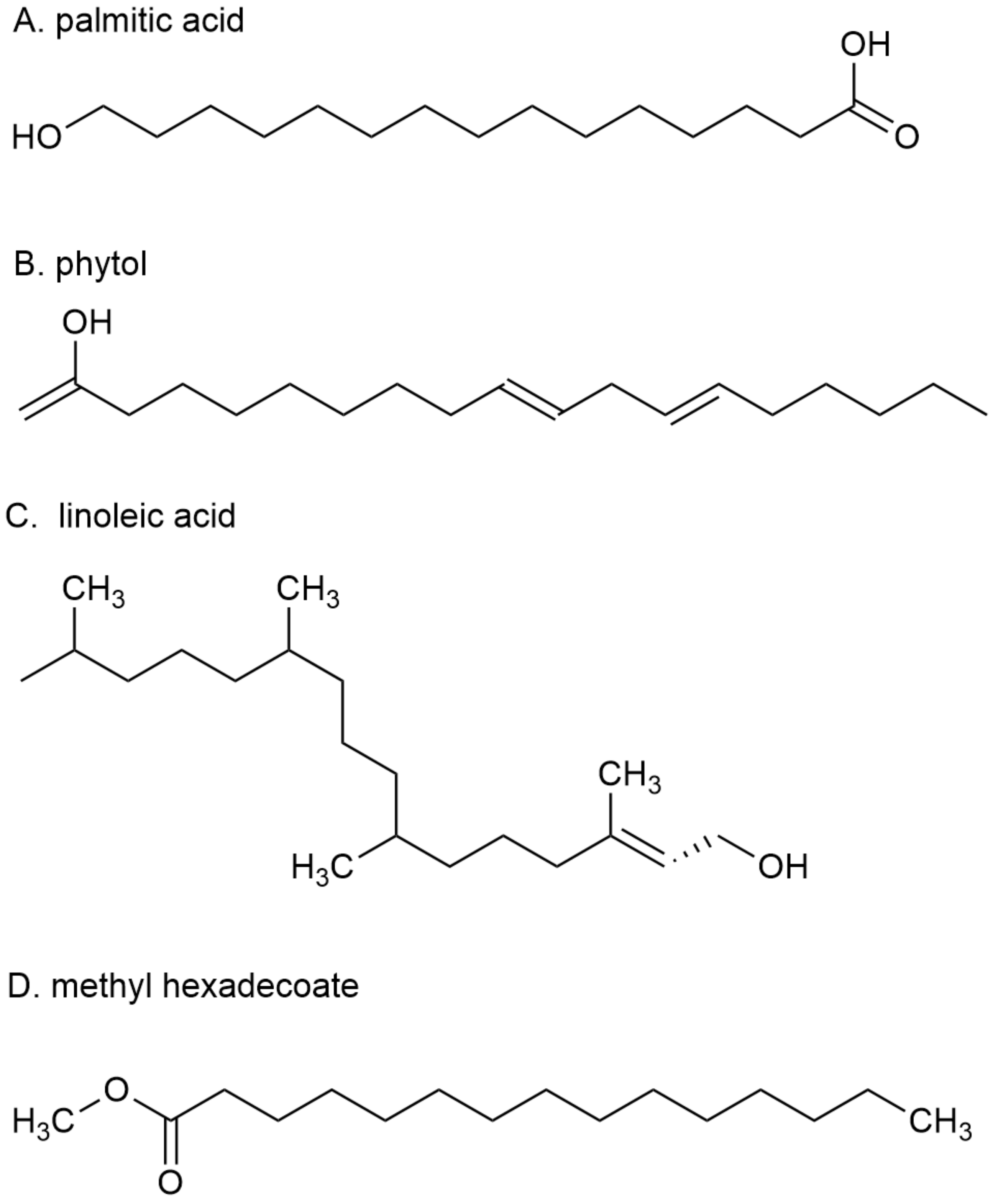

Figure 4. The main metabolites of GVO in stems and leaves.

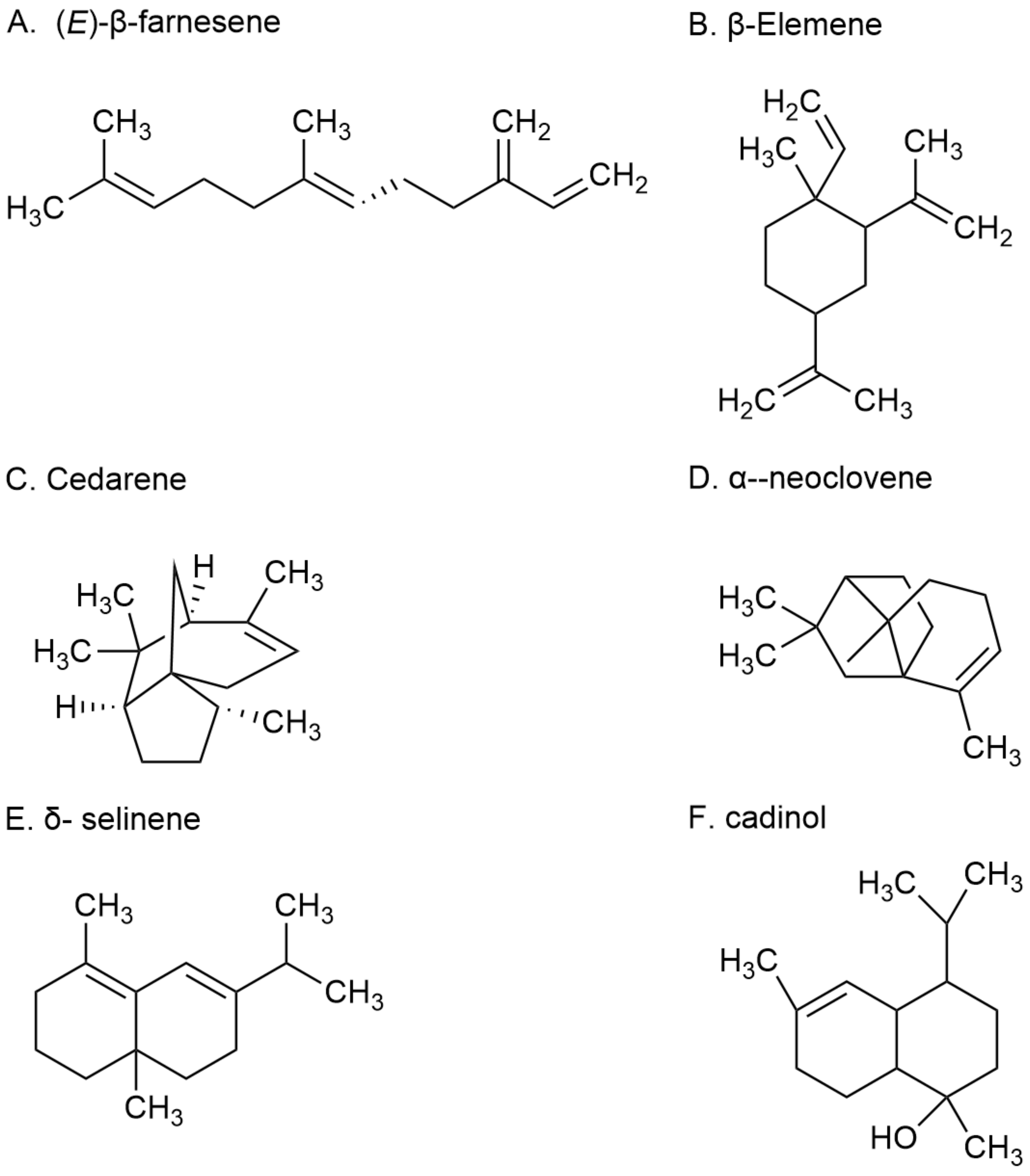

Figure 5. The main metabolites of GVO in fruits.

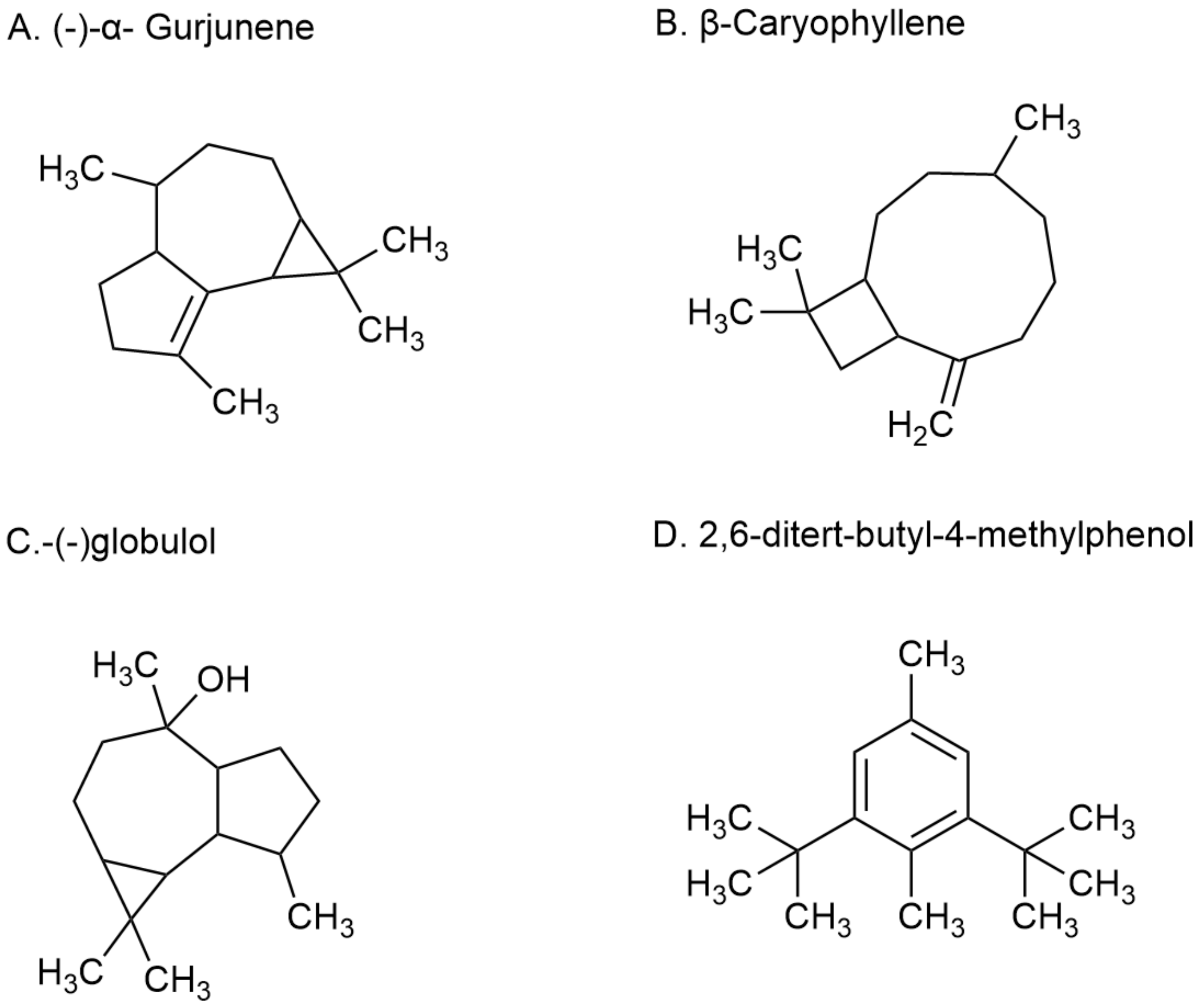

Figure 6. The main metabolites of GVO in rhizomes.

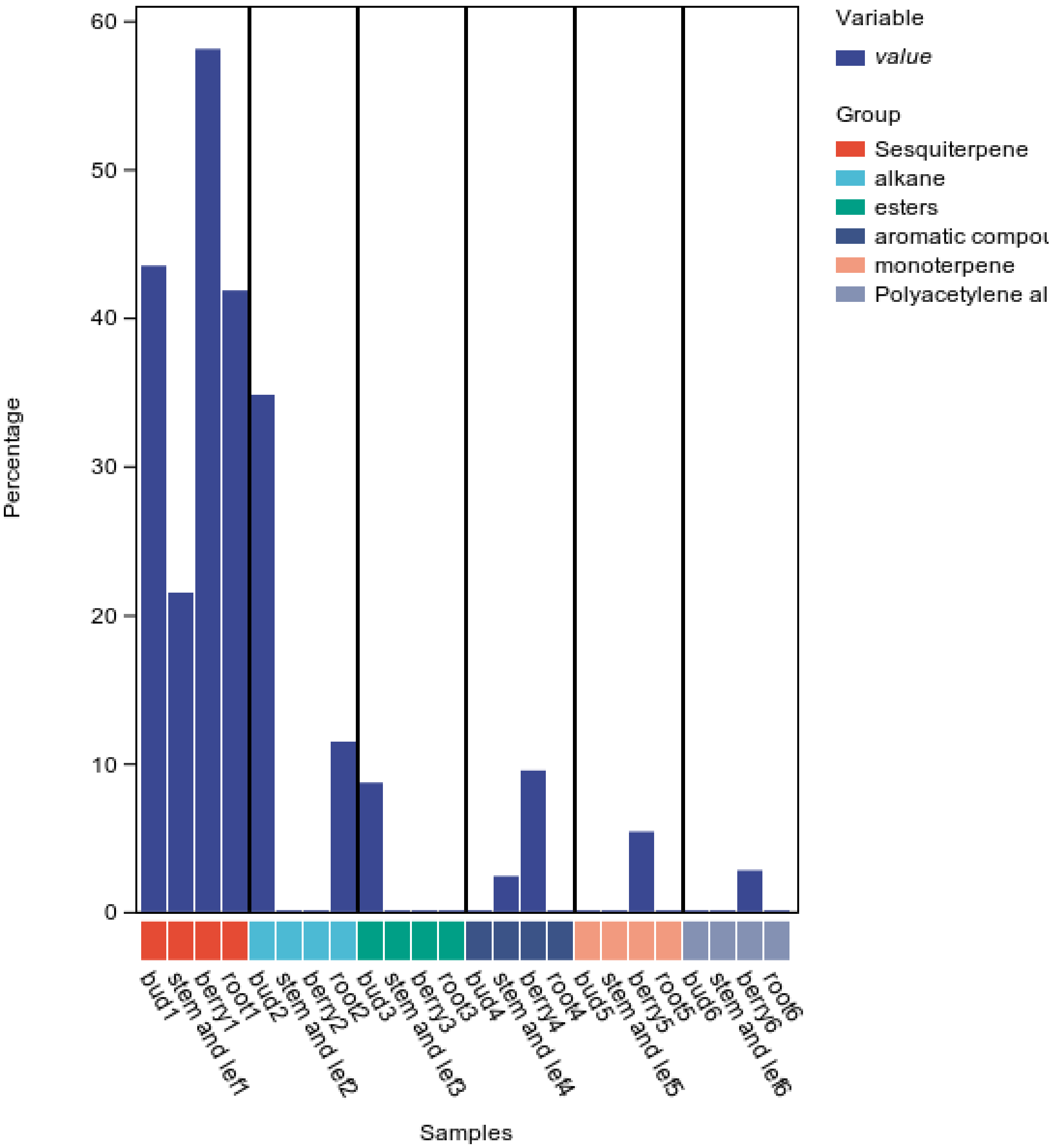

Figure 7. Volatile Oil Content of *P.ginseng* in Different Parts.

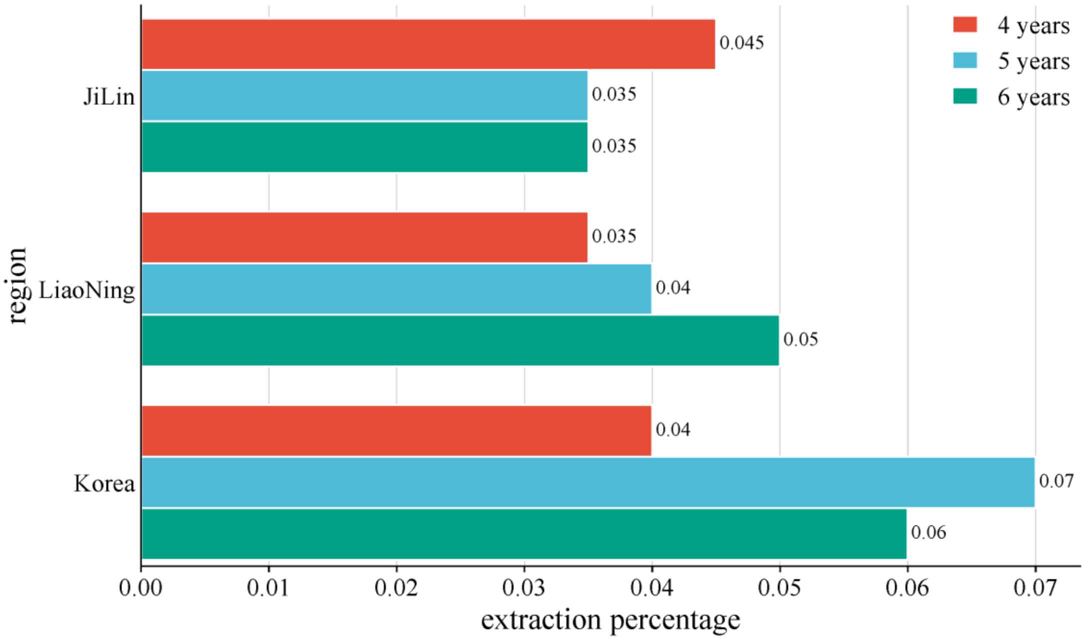

Figure 8. Average yield of GVO by region. The average volatile oil yield of *P.ginseng* from Jilin, Liaoning, and South Korea in China was compared with. It was found that the volatile oil content of four-year-old ginseng: Jilin Province > Liaoning Province > Korea. Five-year-old ginseng: Korea > Liaoning Province > Jilin Province. Six-year-old ginseng: Liaoning Province > Korea > Jilin Province.

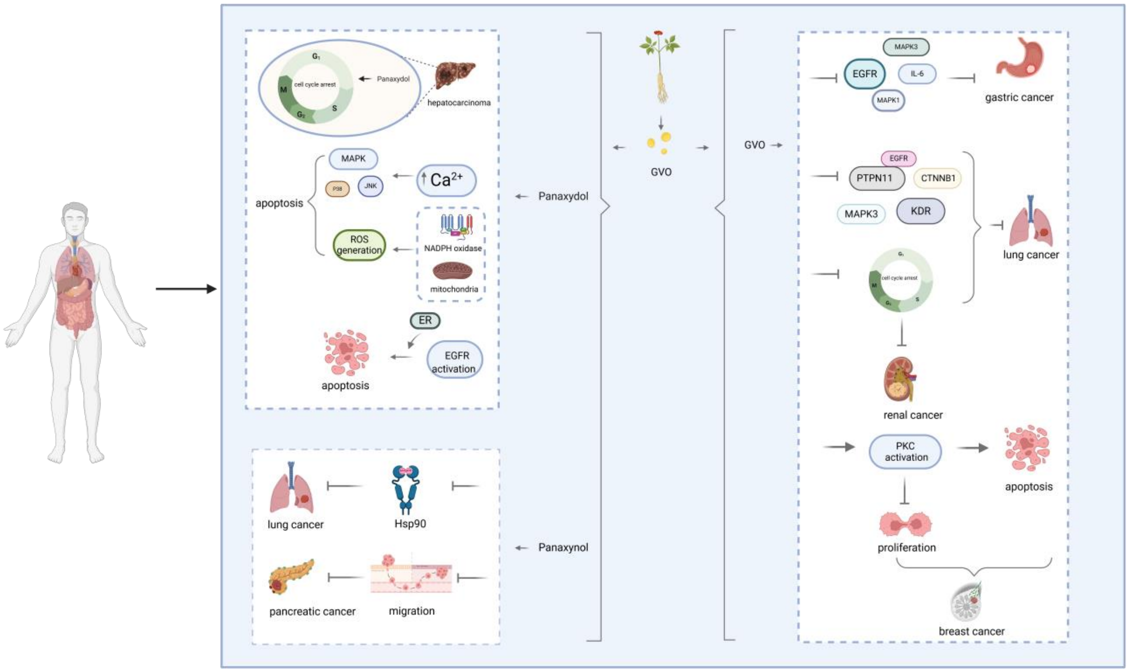

Figure 9. Graphic summary of GVO against cancer.

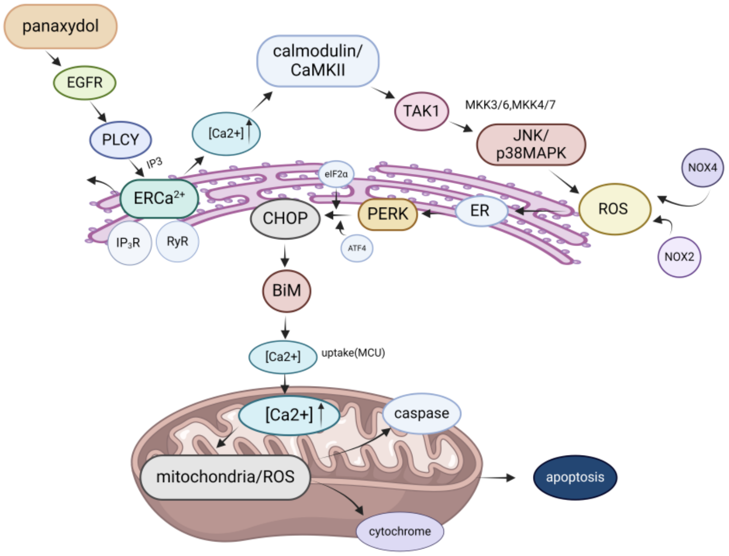

Figure 10. Molecular mechanism of GVO for anti-cancer. The mechanism of Panaxydol induced cell apoptosis involves a rapid increase in cytoplasmic Ca^{2+}concentration, with excess Ca^{2+} transferring from the endoplasmic reticulum (ER) to mitochondria. The release of E［Ca^{2+}］and the resulting increase in $Ca2^{+}$ activate p38 and JNK, while p38/JNK further activates NADPH oxidase. NADPH oxidase activates and induces oxidative stress, triggering mitochondria-dependent apoptosis.

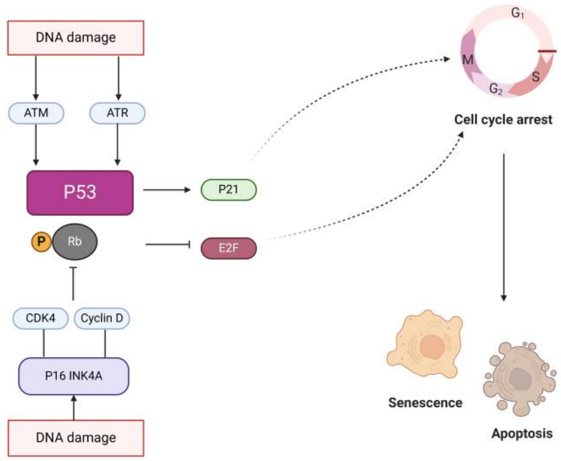

Figure 11. DNA damage-induced senescence pathways.

2 Supplementary Tables

Table 1. Partial metabolites of GVO.

categorization	Metabolite Name	molecular formula	CAS
	β-Guaiene	$C_{15}H_{24}$	88-84-6
	β-patchouline	$C_{15}H_{24}$	514-51-2
Terpenoid	β-farnesene	$C_{15}H_{24}$	18794-84-8
	NNNb-elemene	$C_{15}H_{24}$	251-713-0
	α-cubebene	$C_{15}H_{24}$	17699-14-8
	zingiberene	$C_{15}H_{24}$	495-60-3
	A-Neoclovene	$C_{15}H_{24}$	4545-68-0
	Humulene	$C_{15}H_{24}$	6573-98-6
	Sqalene	$C_{30}H_{50}$	7683-64-9
	caryophyllene	$C_{15}H_{24}$	13877-93-5
	β-caryophllene	$C_{15}H_{24}$	87-44-5
Alcohols	sulfole160	$C_{16}H_{34}S$	25360-09-2
	ergostenol	$C_{28}H_{48}O$	632-32-6
	Chondrillast-7-enol	$C_{29}H_{50}O$	18525-35-4
	hydroxy steroids	$C_{29}H_{48}O$	68555-08-8
	β-sitosterol	$C_{29}H_{50}O$	5779-62-4
	panaxynol	$C_{17}H_{24}O$	81203-57-8
	panaxydol	$C_{17}H_{24}O_2$	72800-72-7
	panaxydiol	$C_{17}H_{24}O_2$	63910-76-9
	(-)-panaxytriol	$C_{17}H_{24}O_3$	87005-03-6
Ketones, aldehydes	β-saccharostenone	$C_{29}H_{46}O$	2034-72-2
	β-sitostenone	$C_{29}H_{48}O$	1058-61-3
	pacoch3	$C_{17}H_{34}O$	2922-51-2
	octylaldehydes	$C_8H_{16}O$	124-13-0
Phenols, heterocyclics	4-ethenyl-2-methoxyphenol	$C_9H_{10}O_2$	7786-61-0
	vita plus E	$C_{29}H_{50}O_2$	59-02-9
	n-hexatricontane	$C_{36}H_{74}$	630-06-8
	octatriacontane	$C_{38}H_{78}$	7194-85-6
	n-henicosane	$C_{21}H_{44}$	629-94-7
	alkane c15	$C_{15}H_{32}$	629-62-9
	1，2-hexadecene epoxide	$C_{16}H_{32}O$	7320-37-8

Table 2. Comparison of different extraction processes for GVO.

**Extraction method**	**extraction rate**	**advantages and disadvantages**
steam distillation	0.3%-0.5%	Advantages: Suitable for samples with high volatile metabolite content, such as plant essential oils.<br>Disadvantages: The ratio of sample to water mixture must be well controlled, otherwise it will affect the separation effect.
reflux extraction	0.1%-0.3%	Advantages: High efficiency. The reflux extraction method requires less reference solvent, is relatively simple to operate, and has higher efficiency.<br>Disadvantages: High solvent consumption.

Ultrasonic extraction technology	0.05%	Advantages: The extraction efficiency is high, and the temperature during the time is low.<br>Disadvantages: Issues such as high container requirements, noise, and equipment amplification.
Supercritical fluid extraction	1.12%	Advantages: High extraction efficiency, recyclable extraction fluid, preventing pollution to the human body and environment during the extraction process.<br>Disadvantages: The recovery rate is influenced by the matrix in the sample; Extracting polar substances requires adding polar solvents and operating under high pressure, resulting in higher equipment investment.

Table 3. The targets and mechanisms of GVO in anti-inflammatory.

Activities	Model	Treatment	Mechanism	References
GVO	Balb/c mice<br>RAW264.7 Cells	Mouse:10%(v/v), 5%(v/v), 1%(v/v)<br>Cells:1μg/mL, 0.8μg/mL, 0.6μg/mL	NF-KB pathway	(Zuo, 2021)
panaxydol	C57BL/6 mice<br>BEAS-2B cells	C57BL/6 mice:20mg/kg panaxydol<br>BEAS-2B cells: 10, 20, 40, and 80 μg/mL	Keap1-Nrf2/HO-1 pathway	(Li et al., 2021)
Chang Shen Hua volatile oil	Male SD rats<br>PC12 cells	Male SD rats :0.1 mL/kg, 0.05 mL/kg<br>PC12 cells : 80μg/mL, 40μg/mL, 20μg/mL	cAMP-PKA-CREB signaling pathway.	(Shuangli et al., 2024)

Table 4. The targets and mechanisms of GVO in antibacterial.

Activities	Disease	Model	Treatment	Mechanism	References
panaxytriol	Helicobacter pylori (HP)	HP ATCC43504 strain	Panaxytriol: 50 microg/ mL.	inhibition	(Bae et al., 2001)
hydrophobic fraction of red ginseng ethanol extracts(Panaxynol and panaxydol)	Acne	*Propionibacterium acnes*<br>female volunteers.<br>men and women age 19 to 40 years	12.5、6.25 and 3.12 mg/mL<br>3 mg/g of RGEF	inhibition	(Contassot et al., 2014)
panaxytriol	Helicobacter pylori	H.Pylori<br>Hela cells	H.Pylori (IC50: 0.05, 0.046 mg/mL)<br>Hela cells: 6mg/mL	inhibition	(Kim et al., 2003)

Table 5. The targets and mechanisms of GVO in anticancer.

Activities	Disease	Model	Treatment	Mechanism	References
hexane extract of ginseng marc (HEGM), hexane-soluble fraction (denoted as HEG)	cancer	Human hepatoma (HepG2) and human breast cancer (MCF-7) cell lines	HEG showed strong inhibition of HepG2 (GI50: 21.1 μg/mL) and MCF-7 (GI50: 41.2 μg/mL.<br>HEGM(GI50: 41.7 μg/mL in HepG2 , GI50: 54.4 μg/mL in MCF-7)	inhibit proliferation	(Lee et al., 2009)

panaxydol	human malignant tumor cell	human melanoma cell line SK-MEL-1	5, 10, 20μg/mL	inhibit cell cycle	(Moon et al., 2000)
petroleum ether extract of panax ginseng roots	cancer	human renal cells carcinoma (RCC) cell lines(Caki-1, A498, and CURC II)	0, 40, 80μg/mL	inhibit cell cycle	(Sohn et al., 1998)
panaxydol	cancer	BEAS-2B (normal immortalized), 1799 (non-transformed), 1198 (transformed but non-tumorigenic) and 1170-I (tumorigenic) cell lines comprising an in vivo lung carci-nogenesis model. human leukemia T cell line, Jurkat, and a human breast cancer cell line, MCF-7,	MCF-7 and Jurkat cells were treated with 50 and 40 μg/mL of panaxydol, BEAS-2B, 1799, 1198 and 1170-I cells were treated with 50 μg/mL panaxydol	increase in $[Ca^{2+}]i$, activation of JNK and p38 MAPK, and ROS generation through NADPH oxidase and mitochondria	(Kim et al., 2011)
panaxydol	cancer	MCF-7 human breast cancer, Pathogen-free female BALB/c or BALB/c nu/nu mice	MCF-7 cells were treated with 20 or 50 μg/mL Mouse: panaxydol (50, 100mg /kg)	EGFR activation and ER stress mediate panaxydol-induced apoptosis. CAMKII-TAK1-p38/JNK signaling pathway	(Kim et al., 2016)
GVO	gastric cancer	MKN-45 cells、HGC-27 cells	MKN-45 cells: 0μg/mL、50μg/mL、100μg/mL. HGC-27 cells：150μg/mL、200μg/mL and 250μg/mL	Regulation of EGFR, MAPK1, MAPK3, and IL-6 related signaling pathways	(Cui, 2022)
lipid-soluble ginseng extract	lung cancer	NCI-H460 human lung cancer cell line	0、30、100μg/mL	cell cycle arrest and apoptosis induction.	(Kang et al., 2011)
fat-soluble metabolites	lung cancer	NCI-H1299 human lung cancer cell line	0、50、100、150、200 and 250 μg/mL）	EGFR、KDR、MAPK3、PTPN1	(Gao et al., 2023)

				1 and CTNNB1 signaling pathway	
panaxynol	lung cancer	human NSCLC cell lines human lung epithelium human retinal pigmental epithelium (RPE),	human NSCLC cell lines: 5 mmol/L. BEAS-2B: <1um , HBE: 1um RPE: 1um	Inhibition of Hsp90	(Le et al., 2018)
panaxynol	pancreatic cancer	Panc-1	80μg/mL	Inhibits the migration and invasion of pancreatic cancer stem cells	(Wang et al., 2015)
P. ginseng seed oil (GSO)	breast cancer	MCF-7 breast cancer cells	1 μL/mL	Attack ability causes apoptosis of ER+ breast cancer cells via PKC activation.	(Kim et al., 2020)
panaxydol	hepatocarcinoma	Human liver carcinoma cell line HepG2	4, 6 and 9 um	blocks the cell cycle progression of HepG2 cells,	(Guo et al., 2009)

Table 6. The targets and mechanisms of GVO in antioxidant.

Activities	Disease	Model	Treatment	Mechanism	References
GVO	extend life	Caenorhabditis elegans strains	12.5、25 and 50 μg/mL	Antioxidant, upregulation of autophagy-related genes atg-4.2, atg-7, lgg-2 and cyd-1	(Wang et al., 2022)
panaxydol	antioxidant	BEAS-2B (normal immortalized), 1799 (non-transformed), 1198 (transformed but non-tumorigenic) and 1170-I (tumorigenic) cell lines	50 μg/mL	$[Ca^{2+}]$i increase, JNK and p38 MAPK activation, and ROS generation through NADPH oxidase and mitochondria.	(Nie et al., 2008)

Table 7. The targets and mechanisms of GVO in neuroprotection.

Activities	Disease	Model	Treatment	Mechanism	References
panaxydol and panaxynol	apoptosis in cortical neurons	pure cortical neurons	5 um	Downregulation of the pro-apoptotic gene Bax and downregulation of the anti-apoptotic gene Bcl-2 were reduced	(Guo et al., 2021)
panaxynol and the acetylenic triol	memory impairment	PC12h cells and Neuro2a cells ddY strain mice	＞2um	affects the neuritogenesis of paraneurons like PC12h and Neuro2a,	(Yamazaki et al., 2001)

Panax ginseng essential oil and Acorus tatarinowii essential oil, Albizia julibrissin flower essential oil	depression	PC12 cells<br>Male SD rats	PC12 cells<br>:<br>20、40、80μg<br>/mL<br>Male SD rats:<br>0.1 mL/kg、<br>0.05 mL/kg	cAMP-PKA-CREB pathway	(Shuangli et al., 2024)
